# N-glycosylation of viral glycoprotein is a novel determinant for the tropism and virulence of highly pathogenic tick-borne bunyaviruses

**DOI:** 10.1371/journal.ppat.1012348

**Published:** 2024-07-15

**Authors:** Masayuki Shimojima, Satoko Sugimoto, Satoshi Taniguchi, Takahiro Maeki, Tomoki Yoshikawa, Takeshi Kurosu, Shigeru Tajima, Chang-Kweng Lim, Hideki Ebihara

**Affiliations:** 1 Department of Virology I, National Institute of Infectious Diseases, Musashimurayama, Tokyo, Japan; 2 Department of Virology I, National Institute of Infectious Diseases, Shinjuku, Tokyo, Japan; University of New Mexico School of Medicine, UNITED STATES OF AMERICA

## Abstract

Severe fever with thrombocytopenia syndrome (SFTS) virus, a tick-borne bunyavirus, causes a severe/fatal disease termed SFTS; however, the viral virulence is not fully understood. The viral non-structural protein, NSs, is the sole known virulence factor. NSs disturbs host innate immune responses and an NSs-mutant SFTS virus causes no disease in an SFTS animal model. The present study reports a novel determinant of viral tropism as well as virulence in animal models, within the glycoprotein (GP) of SFTS virus and an SFTS-related tick-borne bunyavirus. Infection with mutant SFTS viruses lacking the N-linked glycosylation of GP resulted in negligible usage of calcium-dependent lectins in cells, less efficient infection, high susceptibility to a neutralizing antibody, low cytokine production in macrophage-like cells, and reduced virulence in *Ifnar*^-/-^ mice, when compared with wildtype virus. Three SFTS virus-related bunyaviruses had N-glycosylation motifs at similar positions within their GP and a glycan-deficient mutant of Heartland virus showed *in vitro* and *in vivo* phenotypes like those of the SFTS virus. Thus, N-linked glycosylation of viral GP is a novel determinant for the tropism and virulence of SFTS virus and of a related virus. These findings will help us understand the process of severe/fatal diseases caused by tick-borne bunyaviruses.

## Author summary

Ticks sometimes contain small pathogens in their bodies. Because ticks need blood to survive, upon bloodsucking, the pathogens spread to humans as well as animals and cause diseases in some cases. One such pathogen is the bunyavirus, which includes severe fever with thrombocytopenia syndrome virus, and diseases caused by these viruses are often fatal. Most details of bunyavirus-driven diseases are poorly understood, but a viral protein, NSs, was shown to disturb immune responses in humans. This study reports the discovery and characteristics of a novel virulence factor within another bunyaviral glycoprotein. The novel virulence factor is N-glycosylated modification and involves the viral targets for infection. Mutant viruses lacking the modification in the glycoprotein had limited infection potential in cell culture experiments and weakened virulence in animal experiments. Our findings will help us understand bunyavirus-driven diseases and, hopefully, promote countermeasures against the diseases, leading to safer outdoor activities.

## Introduction

The recently identified severe fever with thrombocytopenia syndrome (SFTS) virus, Heartland virus, Guertu virus and Hunter Island virus are tick-borne bunyaviruses belonging to the genus *Bandavirus*, family *Phenuiviridae*, order *Hareavirales* (formerly *Bunyavirales*) [1–5]. Among them, SFTS virus and Heartland virus cause life-threatening diseases in humans. Furthermore, SFTS especially has a high case fatality rate up to 27% [6,7]. Diseases caused by the two remaining viruses have never been reported [4,5]. Because there are currently no approved vaccines or therapeutics against these diseases, a comprehensive understanding of virus-host interactions will enhance the efficient development of countermeasures.

Bites by ticks infected with SFTS virus are a major infection route for humans. After an incubation period, patients become ill with high fever, gastrointestinal symptoms, lymphadenopathy, fatigue, and other symptoms [8–10]. Laboratory tests are performed to test for characteristic findings including leucopenia, thrombocytopenia, and increased liver enzymes in blood [8–11]. The disease accompanies progressive multiple organ failures in severe and fatal cases or undergoes a self-limiting course in survival cases [8–10]. High viremia, hemophagocytic syndrome, and neurological symptoms as well as multiple organ failures are often observed in severe and fatal SFTS cases, although the processes involved are largely unknown [10,12]. Although virus-producing cells during the early phases of SFTS have not been defined [9], monocytes and/or macrophages may be a primary target of the SFTS virus and thus be involved in SFTS pathogenesis [13–17]. These cells have important roles in innate immunity and modulate adaptive immune responses [18]. In a postmortem analysis of fatal cases, viral antigens were frequently found in B cells and macrophages in lymphoid organs, rather than parenchymal cells in non-lymphoid organs [19]. Innate immunity signaling disturbances by SFTS virus infection have been suggested through *in vitro* and *in vivo* experiments and the resultant cytokine/chemokine storm might cause multiple organ failures, hemophagocytic syndrome, and neurological symptoms [8–10,12,20]. Secondary infection of the lung [21,22] might be caused by deficient innate and adaptive immune responses.

The SFTS viral genome is composed of three RNA segments, L, M, and S, encoding RNA-dependent RNA polymerase, glycoprotein (GP), and nuclear protein and non-structural protein (NSs), respectively [8]. GP, as the sole viral envelope protein, plays a major role in attachment and invasion of cells, and therefore is involved in viral tropism. *In vitro*, but not *in vivo*, SFTS virus proliferates well in various adherent cell lines including epithelial kidney Vero and hepatocellular HuH-7 cell lines but not in lymphoid cell lines including Raji (B cell origin) and Jurkat (T cell origin) [23–25]. It has been reported that the expressions of calcium-dependent lectins (C-type lectins, e.g., DC-SIGN, DC-SIGNR, LSECtin) in lymphoid cell lines increased SFTS virus susceptibility, suggesting that SFTS virus infects macrophages via lectins [25–27]. A recent report on the molecular mechanism(s) of B cell infection suggested increased expression levels of C-C motif chemokine receptor 2, CCR2, were involved [28]. Moreover, it is unclear whether the tropism conferred by GP is involved in SFTS pathogenesis. Because recombinant SFTS viruses with deletion or specific point mutations within NSs show reduced virulence in a fatal animal model of *Ifnar*^-/-^ mice, NSs is considered a virulence factor of the SFTS virus, which causes the above-described disturbances in innate immunity signaling [29,30]. Using an aged ferret model, NSs-recombinant viruses were shown to be vaccine candidates [29].

Heartland virus infection causes a disease like SFTS in humans, but the disease severity is mild, and few fatal cases have been reported [7,31,32]. The NSs of the Heartland virus has anti-innate immune activity like that of the SFTS virus [33,34] and Heartland virus lacking NSs had low virulence in a mouse model [35]. Furthermore, Guertu virus NSs inhibited the innate immune response but via a different mechanism to that used by the SFTS virus and Heartland virus [36]. A recent report showed that SFTS virus NSs affected an autophagy-regulating protein, mTOR, resulting in enhanced viral propagation [37].

To help the development of countermeasures against SFTS, we have accumulated fundamental information on the SFTS virus or viral proteins by analyzing mutants obtained after serial passage of the SFTS virus. Through passage in Vero cells, we previously found that an amino acid within GP was involved in the fusogenicity of infected cells [38]. Another serial passage in HeLa demonstrated the presence of a unique mutant with low virulence in animal models. In the present study, we report results from detailed analyses of SFTS viruses with mutations in GP *in vitro* and *in vivo*. Furthermore, we show that the observed phenomena could be adopted to SFTS virus-related viruses. Because the involvement of GP in virulence has not been reported for SFTS virus and related viruses, these findings will enhance our understanding of the tropism and virulence of highly pathogenic tick-borne bunyaviruses as well as SFTS virus to help the development of countermeasures against the viral infectious diseases.

## Results

### Characteristics of passaged SFTS virus

**Fig 1:**
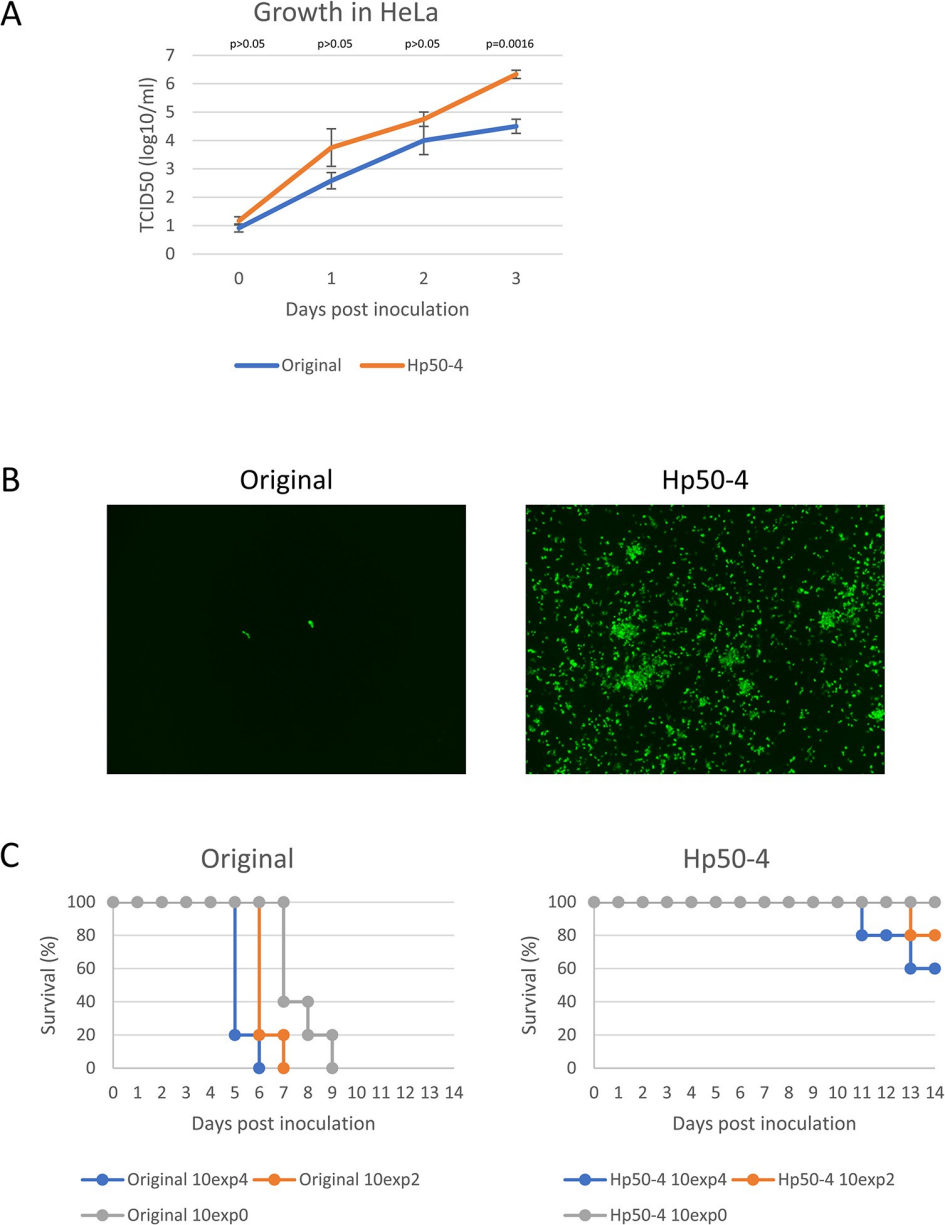
Characteristics of passaged SFTS virus. Confluent monolayers of HeLa cells were inoculated with the original or passaged Hp50-4 strain at a multiplicity of infection of 0.025 and cultured for 3 days. (a) Culture supernatants harvested at the indicated days were titrated in Vero cells. The means and standard deviations are shown (n = 3). Statistical comparisons were performed between the means for each day (Welch’s *t*-test). (b) Inoculated HeLa cells were fixed at 3 days post inoculation and stained with rabbit anti-NP serum. (c) *Ifnar*^-/-^ mice were subcutaneously inoculated with the indicated 50% tissue culture infectious doses of the original or passaged Hp50-4 strain (five mice per group) and observed until 14 days post inoculation. Survival curves are shown.

The passage of SFTS virus SPL030 strain (the original strain) in HeLa cells followed by limiting dilution was performed as described in the Materials & Methods, and a passaged strain, Hp50-4, was obtained. First, the growth kinetics in HeLa cells were compared between the original and Hp50-4 strains. As shown in Fig 1A, Hp50-4 strain had 0.75 to 1.8 log-higher titers than the original at 1 to 3 days post-inoculation (dpi) in supernatants of HeLa cell cultures. When viral antigens in inoculated HeLa cells at 3 dpi were analyzed by indirect fluorescence assay (IFA), higher numbers of viral antigen-positive cells were observed for the Hp50-4 strain than for the original strain (Fig 1B). Next, we used an SFTS mouse model [39] to compare the virulence of the two strains. *Ifnar*^-/-^ mice were subcutaneously inoculated with 10^4^, 10^2^, or 10^0^ tissue culture infectious dose (TCID_50_) of either strain and observed daily for 14 days. The original strain-inoculated mice at any inoculation dose showed a continuous loss of body weight after 3 or 4 dpi, ruffled hair, and reduced activity, and all mice died by 9 dpi (Figs 1C and S1). Among the Hp50-4 strain-inoculated mice, 4 of 5 mice receiving the 10^4^ TCID_50_ inoculation dose and 2 of 5 mice receiving the 10^2^ TCID_50_ inoculation dose showed body weight loss, which started at 9 or 10 dpi (S1 Fig). Among the 6 mice with body weight loss, 3 subsequently developed seizures and died between 10 and 12 dpi whereas the remaining 3 mice recovered. None of the 10^0^ TCID_50_ Hp50-4 virus-inoculated mice showed any symptoms and they died by 14 dpi. Thus, the Hp50-4 strain, which originated from the SFTS virus SPL030 original strain by passaging in HeLa cells, showed slightly more efficient growth in the cell culture and reduced virulence in the SFTS mouse model.

The growth kinetics of the original and Hp50-4 strains were compared in Vero cells (a monkey kidney epithelial cell line), HuH-7 cells (a human hepatoma cell line), and SK-N-SH cells (a human neuroblastoma cell line). As shown in S2 Fig, slightly lower titers of the Hp50-4 strain were observed in Vero and HuH-7 cells at 1 to 3 dpi when compared with those of the original strain. In contrast, no apparent difference was observed between the titers of the two strains in SK-N-SH cells during the observation period (up to 3 dpi, S2 Fig). These data indicated that the passaged Hp50-4 strain adapted to HeLa cells but that their growth properties were not applicable to other cell lines.

### Determinants of Hp50-4 strain phenotypes

**Fig 2:**
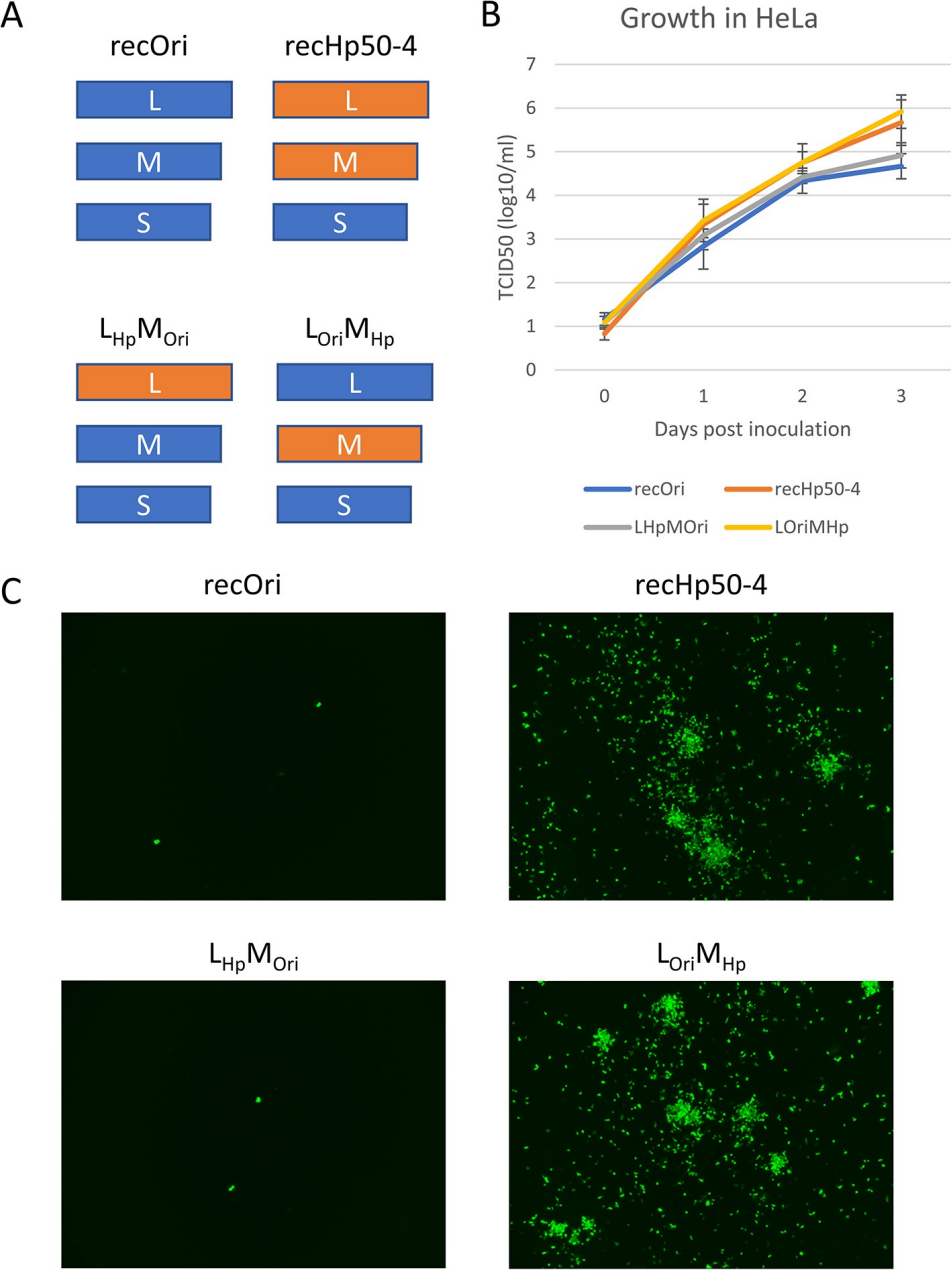
Determinants of Hp50-4 strain phenotypes (*in vitro*). Recombinant viruses were produced using plasmids encoding SFTS viral genomes and used to infect HeLa cells. (a) Schematic segment combinations (L, M, and S) of the produced viruses are shown. Blue and orange squares are from the original and Hp50-4 strains, respectively. Note that the S segment sequence of the Hp50-4 strain was identical to that of the original strain; therefore, the same plasmid for the S segment of the original was used to produce the viruses. (b) Culture supernatants harvested at indicated days were titrated in Vero cells. The means and standard deviations are shown (n = 3). (c) Inoculated HeLa cells were fixed at 3 days post inoculation and stained with rabbit anti-NP serum.

Genome sequences of the Hp50-4 strain were determined by Sanger sequencing and RT-PCR [40] and compared with those of the original strain. Nucleotide differences were observed in the L and M segments, but not in the S segment, of the viral genomes and are summarized in Table 1. No deletions were observed in the Hp50-4 strain genome. Based on the determined sequences, plasmids for the reverse genetics of the SFTS virus [27,41] were prepared and used for the preparation of recombinant viruses (recOri for the original and recHp50-4 for Hp50-4) and chimeric viruses (L_Hp_M_Ori_ whose L and M segments originated from Hp50-4 and the original strains, respectively, and L_Ori_M_Hp_ whose L and M segments originated from the original and Hp50-4 strains, respectively) (Fig 2A). Growth of recOri and recHp50-4 in HeLa cells (Fig 2B) and viral antigen distribution in inoculated cells (Fig 2C) resembled those of the original and Hp50-4 strains, respectively (Fig 1A and 1B). In the experiments, L_Hp_M_Ori_ and L_Ori_M_Hp_ strains were similar to those of the original (or recOri) and Hp50-4 (or recHp50-4) strains, respectively (Fig 2B and 2C), indicating that the changed phenotype observed was attributable to the nucleotide changes within the M segment. Several plasmids for the M segment, which encoded chimeric sequences from the original and Hp50-4 strains, were prepared to produce a panel of recombinant viruses by reverse genetics in which the L and S segments were from the original strain (S3A Fig). IFA with a panel of recombinants was used to show that the nucleotide at position 123 in the M segment (U in the original and A in Hp50-4) affected the growth phenotype of HeLa cells (S3B Fig): the induction of an U to A mutation at position 123 in the background of the recOri strain [recOri(U123A) strain] resulted in a large spread of viral antigens in HeLa cells, whereas the induction of an A to U mutation at position 123 in the background of L_Ori_M_Hp_ strain [recL_Ori_M_Hp_ (A123U) strain] resulted in a limited spread of viral antigens in HeLa cells.

**Table 1:** Nucleotide and amino acid differences between the original and Hp50-4 strains.

Segment	Nucleotide			Amino Acid		
	Position	Original	Hp50-4	Position	Original	Hp50-4
M	123	U	A	35	Ser	Arg
	500	G	A	161	Gly	Glu
	922	G	A	304	Arg	Lys
	1720	C	U	568	His	Tyr
L	391	U	G	125	Val	Val
	547	U	C	177	Val	Val
	1279	G	A	421	Gln	Gln
	1607	G	A	531	Ala	Thr
	1930	U	C	638	Cys	Cys
	3516	C	U	1167	Ser	Leu
	5993	G	A	1993	Val	Met

**Fig 3:**
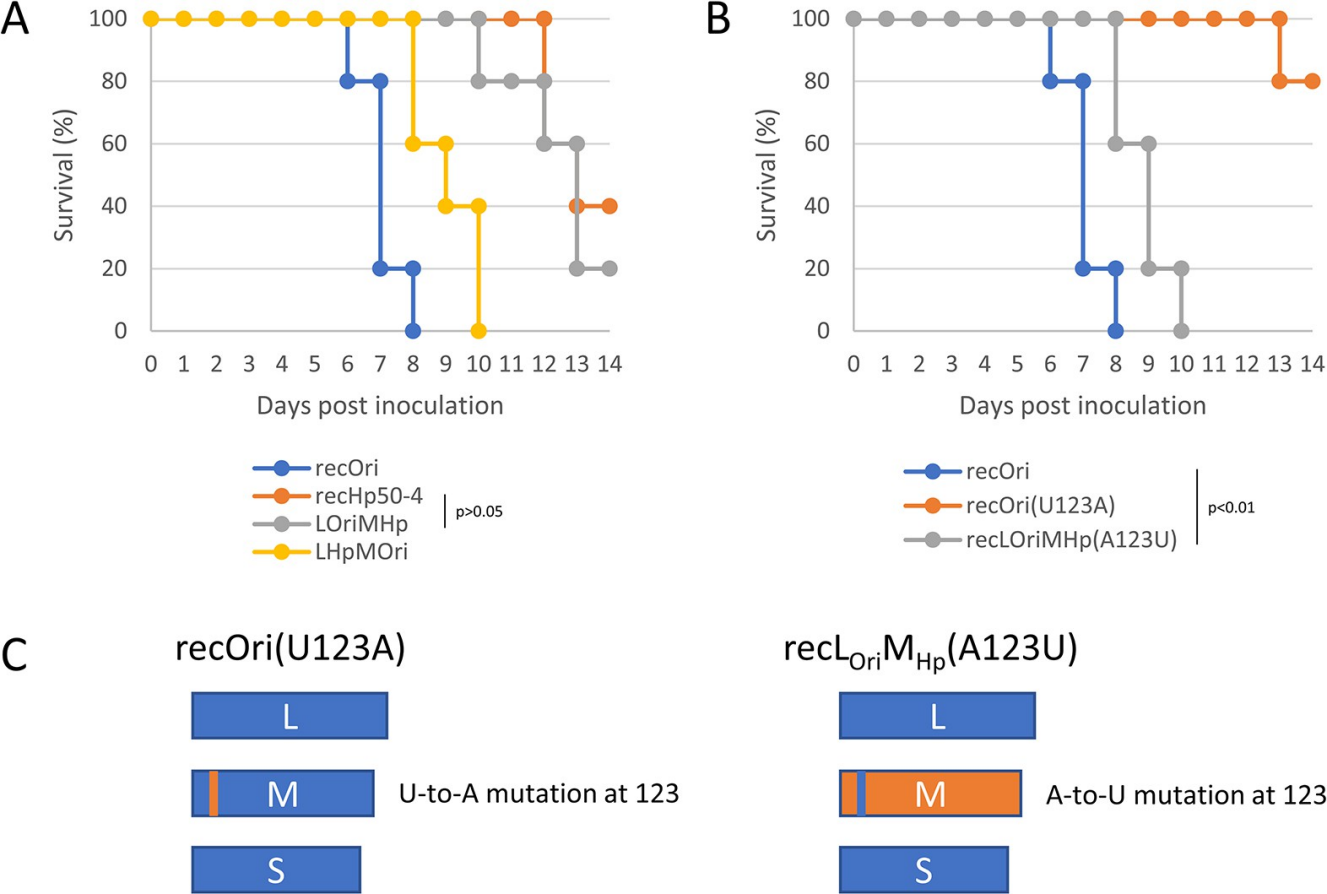
Determinants of Hp50-4 strain phenotypes (*in vivo*). *Ifnar*^-/-^ mice were subcutaneously inoculated with 10^2^ 50% tissue culture infectious doses of recombinant viruses (five mice per group) and observed until 14 days post inoculation. (a)(b) Survival curves are shown. Segment combinations of the recombinant viruses used in (a) are shown in Fig 2A. Schematic diagrams of the genome compositions and point mutations of recOri(U123A) and recLOriMHp(A123U) used in (b) are shown in (c).

Whether the viral factors responsible for the *in vitro* phenotype change (Figs 2 and S3) involved the *in vivo* phenotype changes (Fig 1C) were investigated. For the first step, four recombinants, recOri, recHp50-4, L_Hp_M_Ori_ and L_Ori_M_Hp_ strains were tested at an infection dose of 10^2^ TCID_50_. Mice inoculated with the recOri strain died by 6 to 8 dpi (Fig 3A) and the result was consistent with that of the original strain-inoculated mice (Fig 1C). In contrast, some mice inoculated with the recHp50-4 strain died near the end of the observation period at 14 dpi (Fig 3A), resembling the Hp50-4 strain (Fig 1C). All mice inoculated with the L_Hp_M_Ori_ strain died by 10 dpi, whereas they survived longer than those with recOri (Fig 3A, p<0.01 log-rank test). Some of the L_Ori_M_Hp_ strain-inoculated mice died at 10 dpi or later and the survival time for the strain-inoculated mice was not significantly different from that of the recHp50-4-inoculated mice (Fig 3A, p>0.05 log-rank test). These results indicated that the M segment from the Hp50-4 strain was the major component that affected the phenotype change in the mouse model used, whereas the mutations within the L segment might partially affect the viral virulence. For the second step, three recombinant strains, recOri, recOri(U123A), and recL_Ori_M_Hp_ (A123U) strains were compared in the mouse model. Mice inoculated with the recOri strain died by 6 to 8 dpi as expected, whereas among the 5 mice inoculated with the recOri(U123A) strain, only 1 mouse died at 13 dpi (Fig 3B). All recL_Ori_M_Hp_ (A123U) strain-inoculated mice died between 8 and 10 dpi but they survived longer than those with recOri (Fig 3B, p<0.01 log-rank test). These results indicated that the major part of the observed phenotype change in the mouse model was attributable to the nucleotide difference at position 123 of the M segment (U or A) and that the remaining nucleotide differences in the segment had minor roles. Thus, it was revealed that the same mutation within the M segment (nucleotide position 123) was involved in the growth properties in HeLa cells and virulence in the mouse model.

### Glycosylation status of the GP of SFTS virus strains

**Fig 4:**
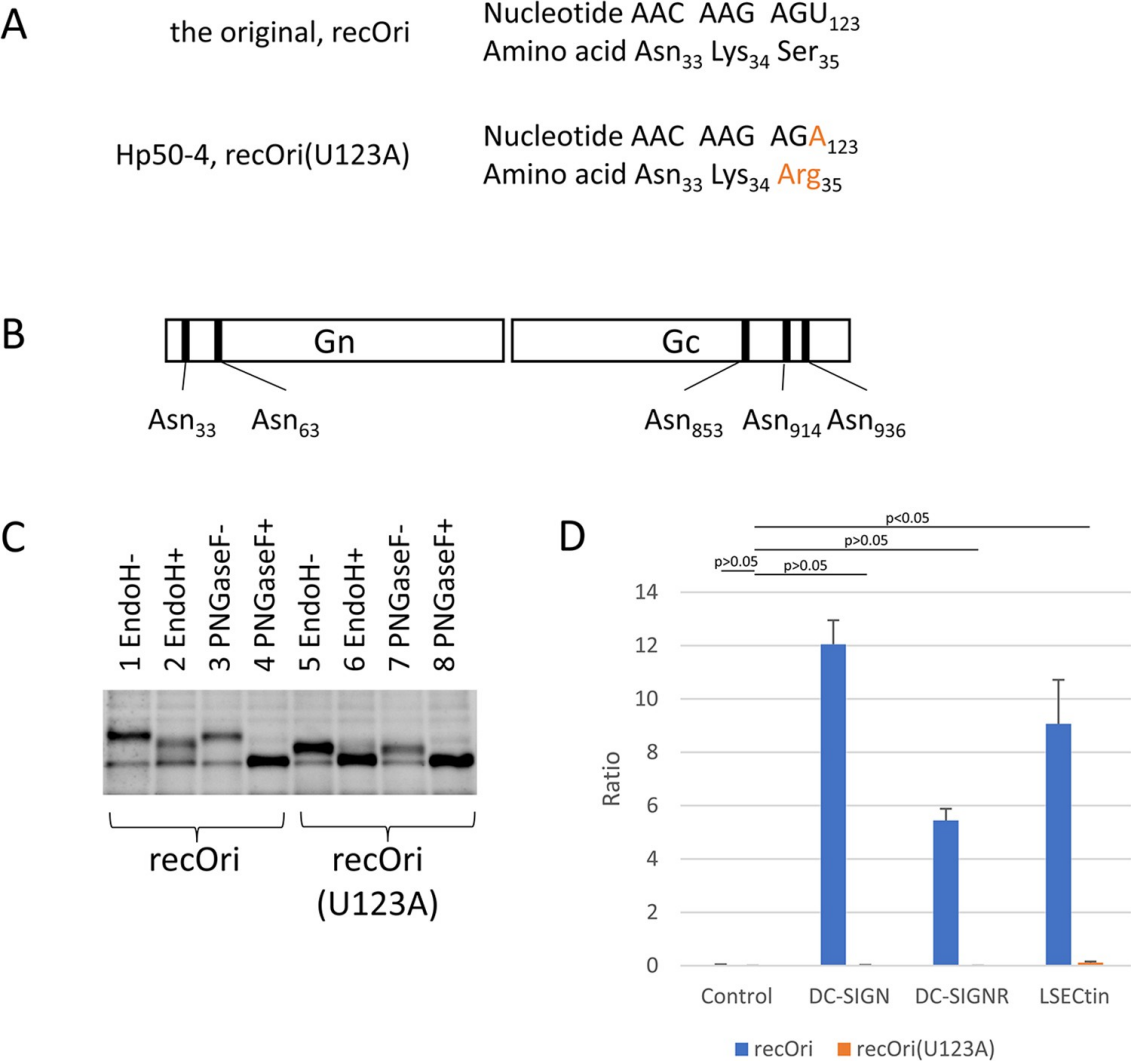
Glycosylation status of the GP of SFTS virus strains and C-type lectin usage upon infection. (a) Nucleotide (position 115 to 123)/amino acid (position 33 to 35) sequences of the original (recOri) and Hp50-4 (recOri(U123A)) M segment/GP are shown. A nucleotide mutation observed in the Hp50-4 strain within the region and deduced amino acids are shown in orange. (b) Schematic diagram of the SFTS virus Gn and Gc with five potential N-linked glycosylation sites (black bars) are shown. (c) Western blotting analysis of lysates of Vero cells infected with recOri or recOri(U123A) recombinants were performed with (+) or without (-) glycosidase treatment and anti-Gn antibody. (d) Jurkat cells expressing a control molecule or one of the human C-type lectins (DC-SIGN, DC-SIGNR, and LSECtin) and Vero cells were inoculated with either recOri or recOri(U123A) strain at an MOI of 0.025. Ratios of viral antigen positivity in Jurkat cells to positivity in Vero cells are shown. Data shown are the means and standard deviations (n = 3). Statistical comparisons were performed between control recOri(U123A) and the others indicated (Dunnett’s test).

The nucleotide mutation U to A at position 123 in the M segment of the SFTS virus, which showed enhanced growth in HeLa cells and reduced virulence in *Ifnar*-/- mice (as described above), had a changed encoded amino acid (aa) at position 35 in GP from serine to arginine (Table 1 and Fig 4A). Because the aa change destroyed the motif for N-linked glycosylation (aa at positions 33, 34 and 35 are asparagine, lysin, and serine, respectively, in the GP of the original virus), the glycosylation status of GP in the recOri and recOri(U123A) strains were examined by western blotting in combination with glycosidases specific to high-mannose, hybrid, and/or complex glycan structures (Endo H and PNGase F). The matured GP of the SFTS virus is composed of two proteins Gn and Gc and it has five potential sites for N-linked glycosylation; two in Gn (1^st^ [position 33] and 2^nd^ [position 63]) and three in Gc (3^rd^ [position 853], 4^th^ [position 914], and 5^th^ [position 936]) (Fig 4B). Because the aa at positions 33 to 35 of GP corresponded to the 1^st^ N-glycosylation motif, an anti-Gn antibody was used as a primary antibody for western blotting to examine the glycosylation status of Gn. Without glycosidase treatment, the Gn of the recOri(U123A) strain migrated more rapidly than that of the recOri strain (Fig 4C, compare lanes 1 and 5 or 3 and 7), suggesting that the 1^st^ potential site was glycosylated in recOri Gn but not in recOri(U123A) Gn. For recOri(U123A) Gn, Endo H treatment resulted in more rapid migration when compared with no glycosidase treatment (Fig 4C, compare lanes 5 and 6) but the same migration level as PNGase F treatment (Fig 4C, compare lanes 6 and 8), indicating that the 2^nd^ potential site had a high-mannose or hybrid N-glycan. For recOri Gn, Endo H treatment resulted in more rapid migration when compared with no glycosidase treatment (Fig 4C, compare lanes 1 and 2) but slower migration than with PNGase F treatment (Fig 4C, compare lanes 2 and 4), indicating that the 1^st^ potential site had a complex N-glycan. Western blotting with anti-Gc antibody revealed the existence of an N-glycan within Gc but no apparent difference between recOri Gc and recOri(U123A) Gc (S4 Fig).

### Usage of C-type lectins

Because SFTS virus has been shown to infect cells expressing calcium-dependent (C-type) lectins more efficiently than non-expressing cells *in vitro* [25–27] and the enhanced infection probably involves interactions between the glycans of GP and C-type lectins, the infectivity of recOri and recOri(U123A) strains to C-type lectin-expressing cells were compared. Jurkat cells expressing a control molecule or one of the human C-type lectins (DC-SIGN, DC-SIGNR, and LSECtin) and Vero cells (for normalization) were inoculated with either strain at a multiplicity of infection (MOI) of 0.025 overnight and viral antigen-positive cells were examined by intracellular staining flow cytometry. The ratios of positivity in Jurkat cells to positivity in Vero cells were calculated and are shown in Fig 4D. Upon inoculation with the recOri strain, the ratios increased for DC-SIGN, DC-SIGNR, and LSECtin when compared with that for a control molecule (0.019 for control vs more than 5 for others). In contrast, upon inoculation with the recOri(U123A) strain, the ratios increased by a small degree only for LSECtin (9.1 for recOri vs 0.12 for recOri(U123A)). The same experiment was repeated with the murine three C-type lectins, SIGNR1, SIGNR3, and LSECtin. As shown in S5 Fig, increased ratios were observed for all of the murine counterparts upon inoculation with recOri. The infection of recOri(U123A) virus was increased only by murine LSECtin but by a small degree (0.65 for recOri vs 0.077 for recOri(U123A)).

The increased infection of C-type lectin-expressing Jurkat cells was examined further with regards to inhibition by specific sugars. To do this simply, we used an infectious virus-like particle (iVLP), which carries an SFTS virus GP as its envelope protein, as a replication-incompetent recombinant SFTS virus that expresses a reporter protein (a fluorescent protein Venus) in infected cells [27]. DC-SIGN- and LSECtin-mediated infection were inhibited dose dependently by mannan and Glc-NAcβ1-2Man, respectively (S6 Fig), suggesting that the increased infection was conferred by the classical interaction between the C-type lectin and specific carbohydrates on the SFTS virus GP.

To investigate the relevance of the 1^st^ to 5^th^ N-glycosylation motifs of GP with regards to C-type lectin-mediated infection, asparagine in the five N-glycosylation motifs was replaced with glutamine to destroy the motifs (Δ1^st^Ngly to Δ5^th^Ngly, details are described in the Methods). For comparison, GP from the recOri(U123A) strain was used. Because of the undetectable infectivity in Vero cells, iVLP with Δ4^th^Ngly GP was not analyzed further (S7 Fig). As expected, either of three human C-type lectin expressions enhanced the infection of iVLP with the GP from the original strain (iVLP-GP(Ori)) and the enhanced degrees diminished for iVLP with the GP from recOri(U123A) (iVLP-GP(U123A)) (S8A Fig). In this assay, the effect of DC-SIGNR expression diminished and that of DC-SIGN and LSECtin expression became reduced for iVLP with GP(Δ1 ^st^Ngly) (S8A Fig). For iVLP with GP(Δ2^nd^Ngly), GP(Δ3^rd^Ngly), and GP(Δ5^th^Ngly), either of the C-type lectins examined showed infection-enhancing effects, whereas the degrees of enhancement varied (S8A Fig). Thus, the enhancing effects of C-type lectins on SFTS virus infection mainly involved the glycan status at aa position 33 (asparagine, the 1^st^ N-glycosylation site) of the viral GP.

Considering the C-type lectin usage of iVLP with GP(Δ1^st^Ngly), the virulence of replication-competent recOri-based mutant whose M segment encoded GP(Δ1^st^Ngly) sequence was examined. *Ifnar*^-/-^ mice were subcutaneously inoculated with 10^2^ TCID_50_ of the recOri or recOri(Δ1^st^Ngly) strain and observed daily for 14 days. As shown in S8B Fig, among four inoculated *Ifnar*^-/-^ mice, only one mouse succumbed to the viral infection at 7 dpi whereas the remaining three survived, indicating that the observed phenotype changes in mutant viruses were not due to aa changes but a lack of N-glycosylation at the 1^st^ N-glycosylation site of GP.

Because the results described above were based on a single strain of SFTS virus (SPL030, genotype J1), we repeated the experiments with other strains, SPL010 (genotype J1) and SPL057 (genotype J3). Reduced C-type lectin usage and reduced virulence in the *Ifnar*^-/-^ mouse model of Δ1^st^Ngly GP-mutant viruses, whose backgrounds were SPL010 and SPL057, were observed (S9 Fig). Thus, the observed results with the SPL030 strain probably reflected the general characteristics of the SFTS virus.

### Involvement of GP N-glycan in SFTS virus infection and cytokine production in macrophage-like cells

**Fig 5:**
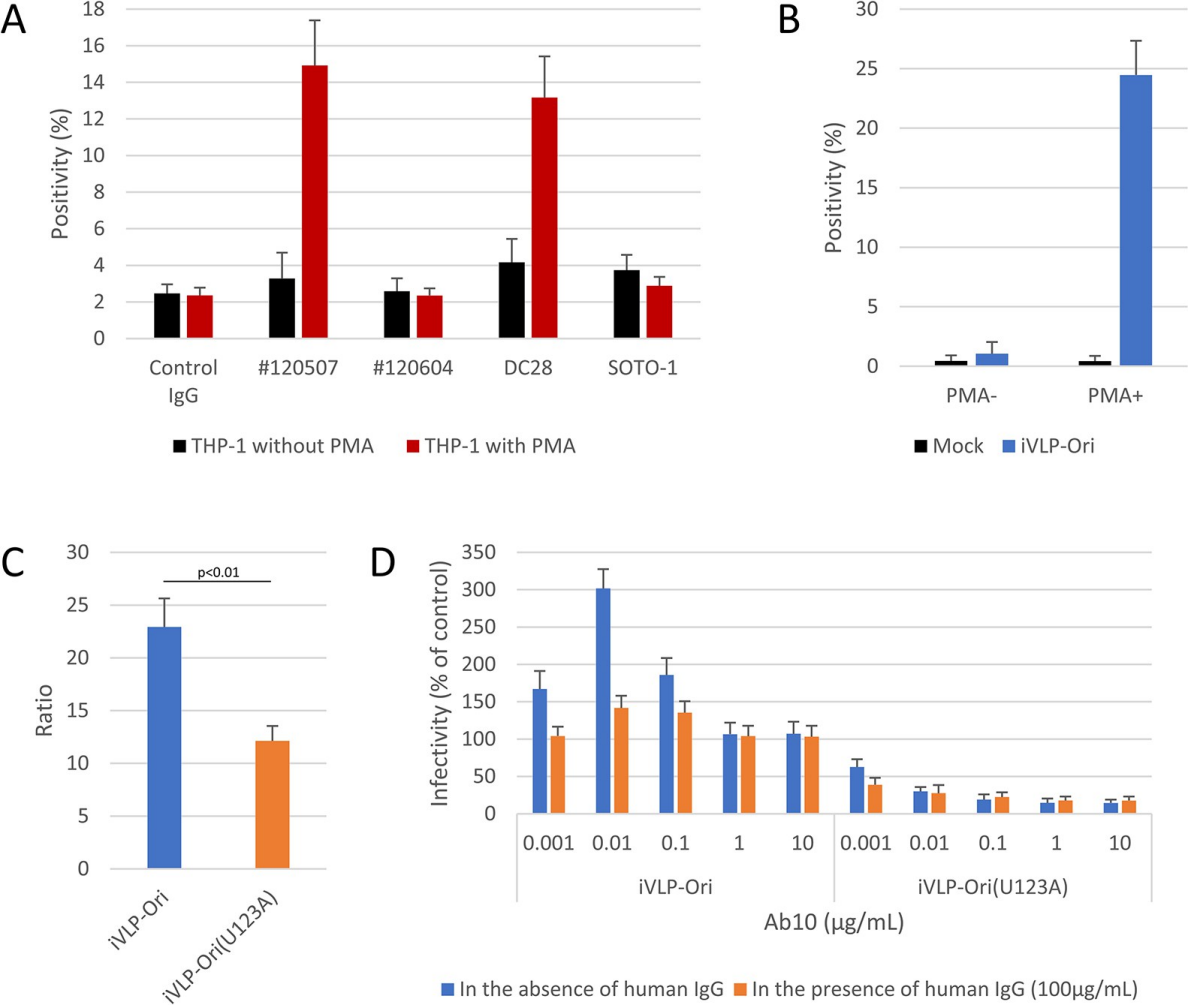
iVLP infection in PMA-treated THP-1 cells. (a) Human monocyte-derived THP-1 cells were cultured in the presence or absence of phorbol 12-myristate 13-acetate (PMA). The expressions of C-type lectins were analyzed by flow cytometry with antibody clones #120507 (DC-SIGN-specific), #120604 (DC-SIGNR-specific), DC28 (DC-SIGN/DC-SIGNR-dual specific), and SOTO-1 (LSECtin-specific). (b) PMA-treated (PMA+) and untreated (PMA-) THP-1 cells were inoculated with infectious viral particle (iVLP) carrying the original GP (iVLP-Ori) and reporter expression was analyzed by flow cytometry (FCM). (c) PMA-treated and untreated THP-1 cells were inoculated with iVLP carrying the GP from the original or recOri(123A) strains (iVLP-Ori or iVLP-Ori(U123A), respectively). Ratios of reporter positivity in treated cells to those in untreated cells are shown. Statistical comparisons were performed (Welch’s *t*-test). (d) PMA-treated THP-1 cells were pretreated or untreated with human IgG followed by the addition of the indicated concentration of Ab10. The cells were further inoculated with iVLP-Ori or iVLP-Ori(U123A). Reporter expression was analyzed by flow cytometry. All data shown are the means and standard deviations (n = 3).

Because C-type lectins are originally expressed in non-T cells including macrophages and dendritic cells and cytokine storm is a characteristic symptom observed in severe/fatal SFTS patients, macrophage-like cells induced from THP-1 cells were used to examine the involvement of GP N-glycosylation in SFTS virus infection. Phorbol 12-myristate 13-acetate (PMA)-treatment increased the expression of DC-SIGN, but not DC-SIGNR nor LSECtin, and enhanced the infection of THP-1 cells by iVLP-GP(Ori) (Fig 5A and 5B). In addition to iVLP-GP(Ori), the PMA treatment of THP-1 cells enhanced the iVLP-GP(U123A) infection, although the increase for iVLP-GP(U123A) was smaller than that of iVLP-GP(Ori) (12.1 vs 22.9, p< 0.01, Fig 5C), suggesting the involvement of the glycan of viral GP in the increasing infection. The pretreatment of PMA-treated cells with a C-type lectin-blocking antibody (clone #120507, clone #120604, and SOTO-1) did not reduce iVLP-GP(Ori) infection and a combination of the three antibodies had no effects (S10A and S10B Fig). Although infection with iVLP-GP(U123A) in PMA-treated THP-1 was inhibited by a human neutralizing monoclonal antibody (clone Ab10 [42]) dose-dependently, the infection of iVLP-GP(Ori) was enhanced by the antibody at 0.001 to 0.1 μg/mL (Fig 5D). The antibody’s enhancing effects were weakened by the pretreatment of cells with 100 μg/mL normal human IgG, but neutralization was not achieved against iVLP-GP(Ori) infection (Fig 5D). This suggested that the SFTS virus infected macrophage-like cells through at least two pathways: one involved the 1^st^ N-glycosylation of GP and was Ab10-resistant and Fc receptor-independent, and the other did not involve the 1^st^ N-glycosylation of GP and was Ab10-sensitive and Fc receptor-dependent.

**Fig 6:**
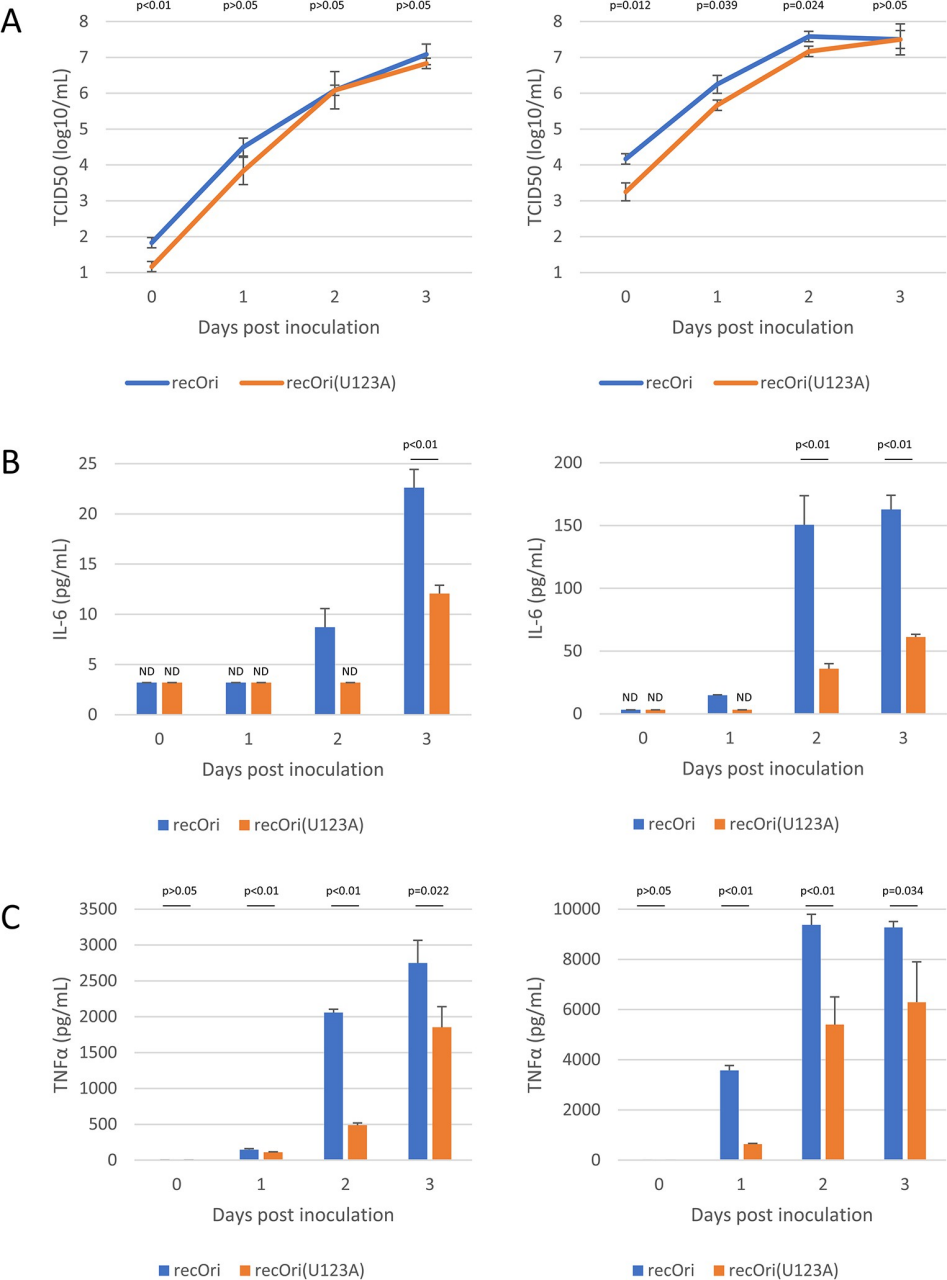
Viral growth and cytokine production in PMA-treated THP-1 cells. Phorbol 12-myristate 13-acetate-treated THP-1 cells were inoculated with recOri or recOri(U123A) at a multiplicity of infection of 0.02 (left) or 2 (right) then cultured for 3 days. Culture supernatants were harvested daily and measured for viral titers (a), IL-6 (b), and TNFα (c). Data shown are the means and standard deviations (n = 3). (a, b, c) Statistical comparisons were performed between the means and p-values are shown (Welch’s *t*-test). ND, not detected. Detection limit for IL-6 (b) was 3.2 pg/mL and bars for ND were set as the detection limit value.

Virus and cytokine production from PMA-treated THP-1 cells were also examined. The cells were inoculated with recOri and recOri(U123A) at low and high MOIs (0.02 and 2) and culture supernatants were harvested daily up to 3 dpi. Viral titers and cytokines (IL-6 and TNFα) in the culture supernatants were measured by ELISA. As shown in Fig 6A, although both viruses grew in the culture during the period examined, the titers of recOri were slightly higher than those of recOri(U123A) only at the early phases (1 and 2 dpi at a high MOI). In contrast, cytokine concentrations were higher in recOri-infected cells than in recOri(U123A)-infected cells at all dpi examined (Fig 6B and 6C). These data indicated that the recOri(U123A) strain had an inefficient or slow induction of cytokine production in macrophage-like cells.

### Analyses of recOri- and recOri(U123A)-infected mice

**Fig 7:**
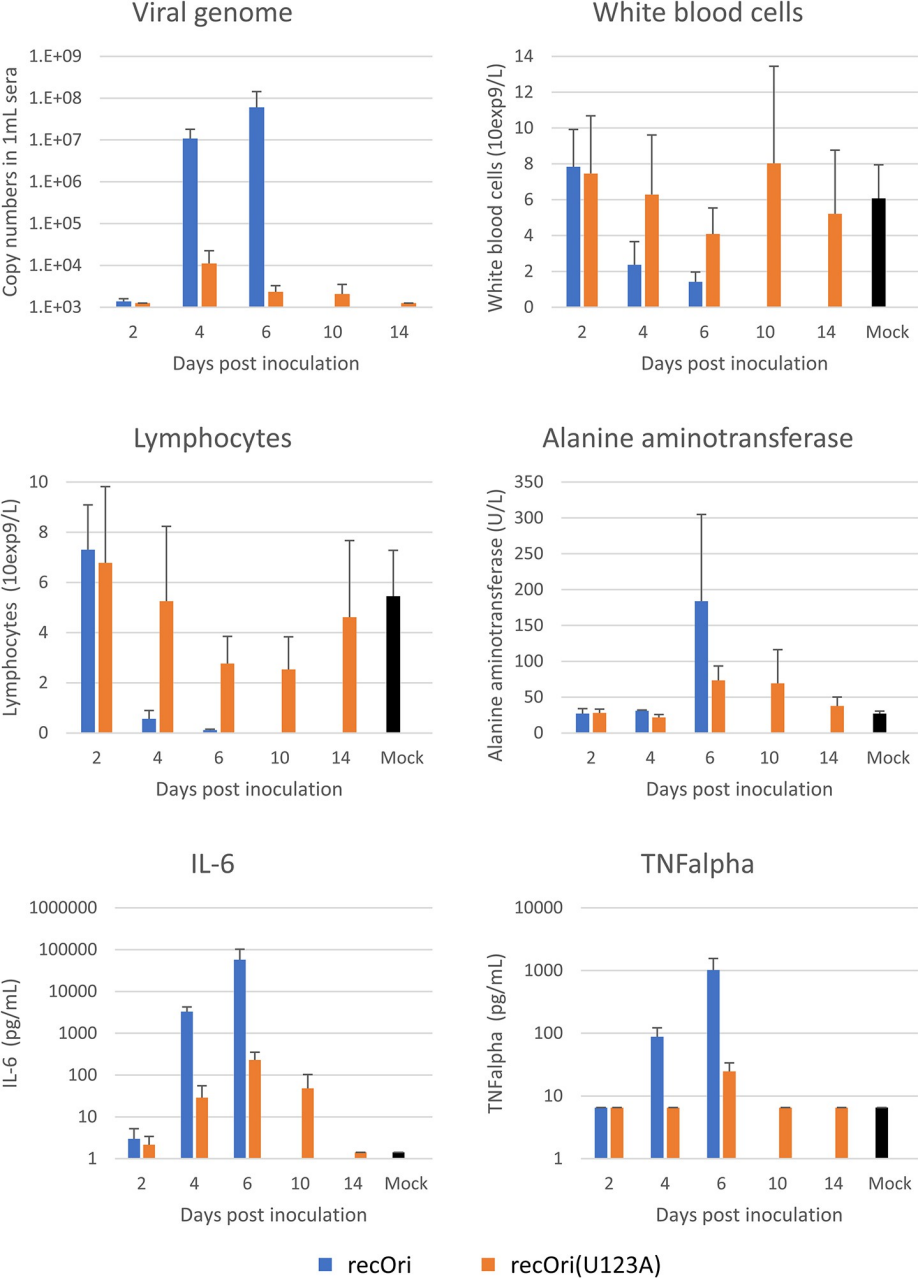
Viral genome distribution and host response in the mouse model. *Ifnar*^-/-^ mice were subcutaneously inoculated with 10^2^ 50% tissue culture infectious doses of recOri or recOri(U123A) and blood sampling was performed upon euthanasia at the indicated days. Sera were used to measure the viral genome copy numbers, alanine aminotransferase, and cytokines. Whole blood was used to measure the numbers of white blood cells and lymphocytes. Blue and orange bars indicate recOri- and recOri(U123A)-infected mice, respectively. Black bars indicate mice injected with control media (6 days post injection). Three mice per group were used at each sampling point.

Previous studies reported high levels of viral genome copies in organs, leukocytopenia, lymphocytopenia, and cytokine/chemokine storm in *Ifnar*^-/-^ mice infected with SFTS virus, rather than lethality [14,42–45]. These features are common clinical features of severe and fatal SFTS patients. In addition, although not examined in the mouse model, the increase of alanine aminotransferase (ALT) and blood urea nitrogen (BUN) and decrease of albumin are often observed in SFTS patients [9,20,46]. Therefore, these characteristics and others were examined in recOri- or recOri(U123A)-infected *Ifnar*^-/-^ mice. Infected mice were euthanized at 2, 4 and 6 dpi [for recOri and recOri(U123A) strains] and at 10 and 14 dpi [for recOri(U123A) strain] to harvest the blood, liver, spleen, kidney, and brain and harvested samples were used for complete blood counting (blood), biochemical analysis (sera), and measurement of cytokines/chemokines (sera) and viral genome copies (sera and organs). In the blood/sera from recOri-infected mice, viremia, leukocytopenia (especially lymphocytopenia), reduction of albumin and glucose levels and increase of globulin levels were observed at 4 dpi and their values worsened at 6 dpi, which was the moribund stage for the infected mice (Figs 7 and S11). Similarly, in the liver, spleen, kidney and brain, the viral genome was detected at 4 dpi and its levels increased at 6 dpi (S11 Fig). Additionally, the increase of ALT, BUN, and cytokines/chemokines (IL-1β, IL-6, TNFα, IL-10, IL-13, IP-10, MCP-1, MIP-1α) were observed in sera at 6 dpi (Figs 7 and S11). In contrast, in recOri(U123A) virus-infected mice, the highest viremia levels were observed at 4 dpi, which were 4-log lower than those in recOri-infected mice (Fig 7). Whereas significant leukocytopenia was not observed, mild lymphocytopenia was observed at 6 and 10 dpi (Fig 7). Although the highest viral genome levels in liver, spleen and kidney were detected at 6 or 10 dpi, the values were quite low when compared with those in recOri-infected mice (S11 Fig). In the brain, however, the highest viral genome levels were similar to those in recOri-infected mice (S11 Fig). Albumin levels in sera were the lowest at 10 dpi (S11 Fig). A moderate increase of globulin and ALT was observed at 10 and 14 dpi, and 6 and 10 dpi, respectively (S11 Fig). Among the cytokines/chemokines, a slight, transient increase was observed only for IL-6 and TNFα (Fig 7). These data indicated that the N-glycan of the SFTS virus GP was involved in the abundant viral replication in various organs, host responses, and host activities in *Ifnar*^-/-^ mice.

To characterize infectivity of SFTS virus replicating *in vivo*, the murine sera harvested at 4 and/or 6 dpi were analyzed regarding infectious titers in Vero cells and infectivity in Jurkat cells. As controls, recOri and recOri (U123A) viruses prepared *in vitro* were diluted and used at 10^5^ TCID_50_/mL. As shown in S12 Fig, recOri virus, but not recOri (U123A) virus, showed higher infectivity in C-type lectin (DC-SIGN, DC-SIGNR, and LSECtin)-expressing Jurkat cells than in control Jurkat cells. Six sera from recOri-infected mice (3 harvested at 4 dpi and 3 harvested at 6 dpi) showed titers of 10^3.75^ to 10^5.5^ TCID_50_/mL in Vero cells and higher infectivity in C-type lectin-expressing Jurkat cells than in control Jurkat cells. Three sera from recOri (U123A)-infected mice (harvested at 4dpi) were also used, because moderate numbers of viral genome copies were detected (Fig 7). However, no detectable titers (<10^2.75^ TCID_50_/mL) or no apparent infectivity in Jurkat cells were observed (S12 Fig). These data suggested that SFTS virus replicating *in vivo* had the ability to infect C-type lectin-expressing cells via N-glycosylated GP.

### N-glycosylation in the GPs of different SFTS virus strains and related viruses

We examined whether the N-linked glycosylation motif observed at aa positions 33 to 35 within the GP of SPL030, SPL010, and SPL057 strains of the SFTS virus was conserved among other strains. Any SFTS virus strains whose sequences corresponded to the aa region and were deposited in GenBank had an identical aa sequence at the regions within the GP, except for the SPL125A strain whose genome sequence was determined by NGS and aa sequence corresponding to the region was a non-N-glycosylation motif (lysine-glutamine-serine). However, Sanger sequencing following RT-PCR with patient RNA prepared independently from the NGS revealed that the aa sequence corresponding to the region in SPL125A was asparagine-lysine-serine, identical to that of the original SPL030 strain. In addition, the other four N-linked glycosylation motifs (2^nd^ to 5^th^) were completely conserved in all SFTS virus strains whose sequences corresponding to each region were deposited. As the genome/aa sequences among SFTS virus strains are highly conserved (over 97% homology within GP aa sequences [47]), these results indicated that the SFTS virus intrinsically has five N-linked glycosylation motifs at the same positions in the GP.

The SFTS virus (Dabie bandavirus) belongs to the Genus *Bandavirus*, which includes eight species: Dabie, Bhanja, Guertu, Heartland, Hunter Island, Kismaayo, Lone Star, and Razdan bandaviruses. Among the eight species, SFTS virus, Guertu bandavirus, and Heartland bandavirus are located close to each other in phylogenetic trees [4] and Hunter Island bandavirus is located slightly further away from the three viruses. Furthermore, the SFTS virus and Heartland bandavirus cause similar diseases in humans, although the latter is comparatively mild [7,31,32]. As shown in S13 Fig, the five N-glycosylation motifs observed in the SFTS virus are completely conserved in the Guertu, Heartland, and Hunter Island bandaviruses, whereas the Hunter Island bandavirus has two additional N-glycosylation motifs (S13 Fig).

### Virulence and usage of C-type lectins in a Heartland bandavirus mutant lacking the 1^st^ N-glycosylation motif

**Fig 8:**
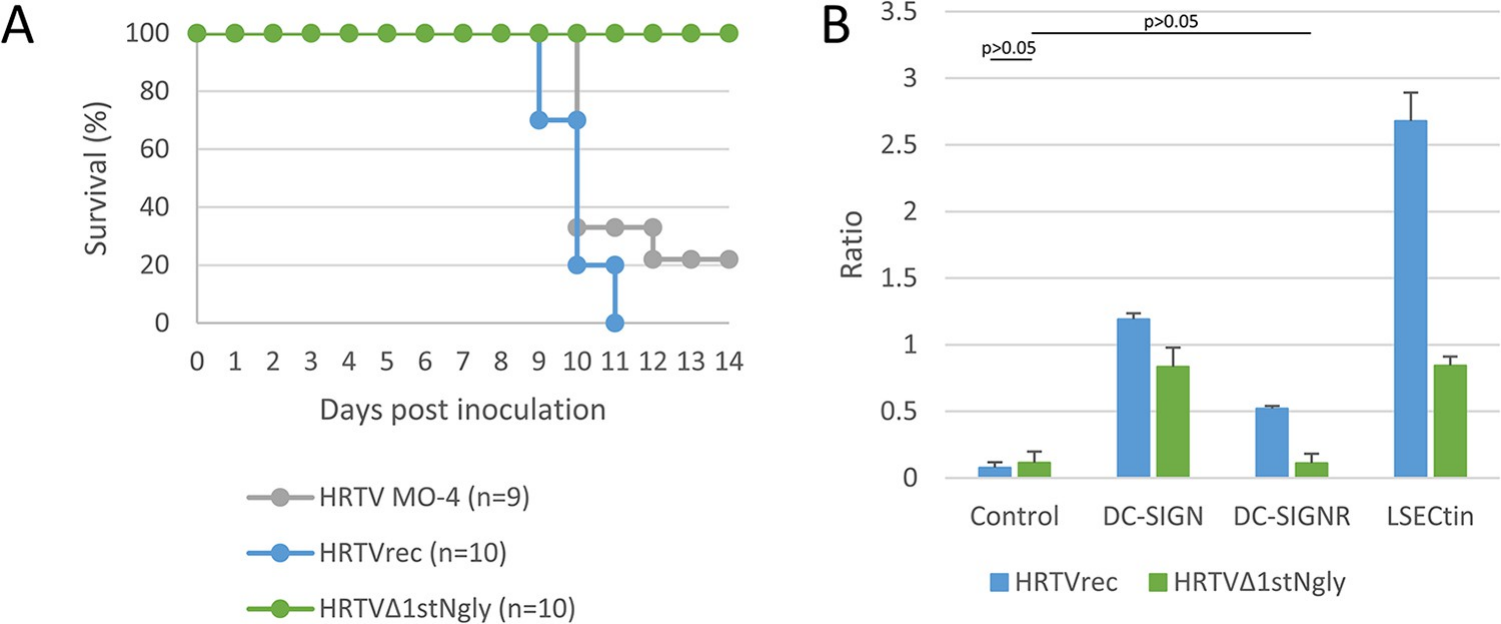
C-type lectin usage and virulence of Heartland virus and recombinants. Based on the genome sequence of the Heartland virus (HRTV) MO-4-NIID strain, two HRTV recombinants, HRTVrec and HRTVΔ1stNgly, were produced by reverse genetics. HRTVrec had no mutation but HRTVΔ1stNgly had an asparagine-to-glutamine substitution at position 35 of GP, resulting in the destruction of the N-linked glycosylation motif. (a) AG129 mice were subcutaneously inoculated with 10^2^ focus forming units of HRTV MO4, HRTVrec, and HRTVΔ1stNgly (nine or ten mice per group) and observed for 14 days. Survival curves are shown. (b) Jurkat cells expressing control molecule or one of the human C-type lectins (DC-SIGN, DC-SIGNR, and LSECtin) and Vero cells were inoculated at a multiplicity of infection of 0.025. Ratios of viral antigen positivity in Jurkat cells to viral antigen positivity in Vero cells are shown. Data shown are the means and standard deviations (n = 3). Statistical comparisons were performed between control HRTVΔ1stNgly and the others indicated (Dunnett’s test).

The Heartland bandavirus infection is lethal for AG129 mice, which genetically lack both *Ifnar* and *Ifngr* genes [35,48]. Because the SFTS virus and Heartland bandavirus cause similar diseases in humans [6,7] and the numbers and sites of N-glycosylation motifs within the GP are highly conserved (as described above), we expected that a lack of a 1^st^ N-glycosylation motif corresponding to that of the SFTS virus would result in the reduced virulence of the Heartland bandavirus. By using reverse genetics recently reported for this virus [35], we prepared two recombinant viruses based on the MO-4-NIID strain, one without a mutation (HRTVrec) and the other having an asparagine-to-glutamine mutation to destroy the motif (HRTVΔ1stNgly). Then, we inoculated them into AG129 mice subcutaneously at 10^2^ focus-forming units per mouse. The parent MO-4-NIID and HRTVrec strains caused body weight loss at 8 dpi and later (S14 Fig). Eight of nine mice inoculated with the MO-4-NIID strain died by 12 dpi and all 10 mice inoculated with HRTVrec died by 11 dpi (Fig 8A). In contrast, none of the 10 mice inoculated with the HRTVΔ1stNgly mutant died until 14 dpi. These results indicated that the virulence of the Heartland virus in AG129 mice could be affected by the deletion of N-glycosylation within Gn.

The usage of the C-type lectins of HRTVrec and HRTVΔ1stNgly were compared. Jurkat cells expressing a control molecule or one of the human C-type lectins and Vero cells were inoculated with either strain at an MOI of 0.025 overnight and viral antigen-positive cells were examined. Ratios of positivity in Jurkat cells to positivity in Vero cells were calculated and are shown in Fig 8B. Increased infectivity ratios of HRTVrec were observed by the expression of DC-SIGN, DC-SIGNR, and LSECtin. Increased ratios of HRTVΔ1stNgly were also observed by DC-SIGN and LSECtin but not by DC-SIGNR expression. The increased ratios observed for HRTVΔ1stNgly were lower than those for HRTVrec.

## Discussion

In this study, *in vitro* and *in vivo* analyses showed that the N-linked glycosylation of the viral GP is a novel tropism and virulence factor of the SFTS virus. The lack of N-linked glycosylation at a specific residue of GP, aa position 33, reduced the ability of the SFTS virus to use C-type lectins upon infection leading to the inefficient infection of macrophage-like cells *in vitro*. This also resulted in increased viral susceptibility to a neutralizing antibody and low-level cytokine production in macrophage-like cells. In mice inoculated with mutant viruses lacking the N-linked glycosylation, the viral burdens and cytokine/chemokine levels in organs and blood were reduced, and the survival time was prolonged or fatality rates were decreased when compared with non-mutant viruses. Based on these findings, we propose a key characteristic during SFTS disease whereby the SFTS virus infects specific cells including monocytes and macrophages via the N-linked glycan of GP in early disease phases. This induces cytokine/chemokine production in the cells, eventually leading to high viremia and cytokine storm. Blockage of the N-linked glycosylation GP-mediated infection and/or cytokine/chemokine production might be a target candidate for the development of therapeutics against SFTS or post-exposure prophylaxis against exposure to the SFTS virus.

The SFTS virus and HRTV genome encode a non-structural protein, NSs. This protein disturbs innate immune responses via many pathways [49, 50] and, probably therefore, modulates the host condition in which the virus efficiently proliferates, and cytokines/chemokines are produced uncontrollably. Mutant viruses lacking NSs or having point mutations in NSs have low virulence in animal models [30,35]. Therefore, the NSs protein is considered a virulence factor of both viruses. N-linked glycosylation mutant viruses, which were examined in the present study, had intact NSs but had low virulence in animal models. Therefore, our study implies that, for NSs to function as a virulence factor, the viruses need to infect specific cells such as those infected by wild SFTS virus, but not mutant viruses lacking N-glycan GP, which prefers to infect monocytes/macrophages. In this view, the N-linked glycosylation in GP reported in the present study might should be called as a virulence-affecting factor, but not a virulence factor. Regarding the reversion of virulence in NSs-mutants [29], double mutations in NSs and GP can make the attenuated property of viruses more stable than a single mutation.

We have no direct evidence showing that C-type lectin-mediated infection is involved in the pathogenicity caused by the SFTS virus. Mice have a homologue of the human LSECtin but no functional homologue of human DC-SGIN or DC-SIGNR [51]. In addition to mouse LSECtin, we selected the mouse C-type lectins, SIGN-R1 and SIGN-R3, which have functions and tissue distributions similar with those of human DC-SIGN/DC-SIGNR [52] to examine the relationship between the N-linked glycosylation of SFTS virus GP and mouse C-type lectins *in vitro*. Because several other mouse C-type lectins partially resemble the human DC-SIGN/DC-SIGNR functions and/or tissue distributions, there is a possibility that such yet unexamined C-type lectin molecule(s) will enhance SFTS virus infection and be involved in SFTS virus-induced disease *in vivo*. Furthermore, blocking all mouse C-type lectins by antibodies and the knock-out of all C-type lectin genes to clarify their significance in SFTS virus infection in mice is unlikely to be feasible and there is no reagent that targets the N-linked glycosylation of SFTS virus GP to inhibit infection. Therefore, in the present study we could not directly examine the C-type lectin involvement in SFTS disease. However, inhibitors that block N-linked glycosylation-mediated stimulation and/or infection, if developed, or pharmacological substances modulating glycosylation pathways will be useful tools to examine the involvement of N-linked glycosylation in the pathogenesis of SFTS disease and might also be a usable therapeutic agent for SFTS treatment.

Efficient infection of SFTS virus in C-type lectin-expressing cells has been reported [25–27]. A recent report showed that a C-C chemokine receptor, CCR2, mediates SFTS virus binding and infection to cells and involves SFTS pathogenesis in a murine model [28]. While mutant SFTS viruses lacking N-linked glycosylation of GP showed negligible usage of C-type lectins in infection and reduced virulence in a murine model (the present study), it is not yet addressed whether the mutant viruses use CCR2. Nevertheless, because cell-type distributions of C-type lectins and CCR2 are partially overlapped, SFTS virus infection in cells expressing both molecules (*e*.*g*., macrophages) might have a significant impact on SFTS pathogenesis. Further studies involving these molecules and mutant viruses are necessary to clarify the cell entry mechanism of SFTS virus and the disease process.

Because N-linked glycosylation is highly conserved among SFTSV/HRTV group members, the N-linked glycosylation of GP (five sites) in these five members might have common and/or fundamental roles in the pathogenesis of disease. In fact, we observed that Heartland virus lacking the 1^st^ N-glycosylation in GP showed a reduced usage of C-type lectins upon infection and became attenuated in a mouse model, similar to the SFTS virus. Therefore, our findings reported in the present study might be applicable to other members of the viral group. Although an infectious disease caused by Guertu virus, a member of the SFTSV/HRTV group, has never been reported, the conserved N-glycosylation of the viral GP as well as anti-innate immunity functions of the viral NSs [36] supports the idea that the virus has the potential to cause disease in humans [4]. The roles of the remaining four N-glycosylation sites of GP are unclear; however, infectious iVLP lacking the 4^th^ N-linked glycosylation of GP was not obtained (S7 Fig) and replication-competent viruses with the mutation could not be obtained by multiple trials of reverse genetics. A recent study by Du et al. [53] reported that the 4^th^ N-linked glycosylation plays crucial roles in the inter-peplomer interactions of GP and infectivity of lentivirus-based pseudoviruses bearing GP. Our results reinforce the findings of Du et al. [53], through the usage of VLP whose backbone is the SFTS virus itself.

Considering SFTS viral antigen positivity in the original strain-inoculated HeLa cells and its supernatant titers determined using Vero cells (Fig 1A and 1B), progeny from HeLa cells seem to have quite low infectivity in HeLa cells. The molecular mechanism(s) by which HeLa-derived virus lacking the 1^st^ N-glycosylation site within GP shows improved infectivity in HeLa cells is unclear. The inefficient proliferation of the SFTS virus in HeLa cells is consistent with previous reports [25,26,54] and different viral infectivity depending on producer cells resemble cases of flaviviruses [55–57], probably making our understanding of the *in vivo* tropism of SFTS virus complicated. Whereas in the present study we focused on the investigation of the attenuation mechanism(s) of the passaged SFTS virus strain, studies on the infection mechanisms in various cell types and on the infectivity of SFTS virus produced from various cell types would help us understand the viral dynamics in SFTS patients.

The pathogenic mechanisms of SFTS have not been completely resolved. In the present study, we showed that the N-linked glycosylation of the viral GP is involved in viral tropism and virulence. Because viral NSs were regarded as the sole virulence factor of the SFTS virus, this is the first report to show involvement of GP with the viral virulence. We hope that our findings will lead to the elucidation of SFTS pathogenesis and eventually to the development of appropriate therapy and efficient vaccination against the disease.

## Materials and methods

### Ethics statement

Experiments with animals were performed in strict accordance with the Animal Experimentation Guidelines of the National Institute of Infectious Diseases. The protocol of animal experiments was approved by the Institutional Animal Care and Use Committee of the National Institute of Infectious Diseases (nos. 118121, 120121, and 121055).

### Viruses

Strains of SFTS virus, SPL030 (genotype J1), SPL010 (J1), and SPL057 (J3) were propagated in Vero cells once and used as the original of each strain. Titration was performed as described previously [38]. The HRTV MO-4-NIID strain was propagated and titrated as described previously [35].

### Cells

Vero, HeLa, HuH-7, Jurkat, and THP-1 cells were obtained from the American Type Culture Collection (Summit Pharmaceuticals International Corporation, Japan). SK-N-SH cells were kindly provided by Prof. Takegami, Kanazawa Medical University. Vero, HeLa, HuH-7, and SK-N-SH cells were cultured in DMEM (Sigma-Aldrich) supplemented with 5% heat-inactivated fetal calf serum (FCS) and antibiotics (Gibco, Pen Strep). Upon inoculation, these cells were maintained in DMEM with 2% FCS (DMEM-2FCS) and antibiotics. Jurkat cells were cultured in RPMI-1640 (Sigma-Aldrich) supplemented with 10% FCS and antibiotics. THP-1 cells were cultured in RPMI-1640 supplemented with 10% FCS, 0.05 mM 2-mercaptoethanol and antibiotics. Stable expressions of C-type lectins in Jurkat cells were performed as described previously [58].

### Antibodies and other reagents

Normal human IgG, rabbit antibody against SFTS virus Gc, and anti-LSECtin (SOTO-1) were purchased from FUJIFILM Wako Pure Chemical Corp., ProSci-Inc (Cat. No. 6653), and Santa Cruz Biotechnology, respectively. PMA and Mannan and GlcNAcβ1-2Man disaccharide were purchased from Sigma-Aldrich and Dextra Laboratories, respectively. Mouse IgG1 isotype control, anti-DC-SIGN (#120507), anti-DC-SIGNR (#120604), and anti-DC-SIGN/DC-SIGNR (DC28) were purchased from R&D Systems (Minneapolis, MN, USA). Human monoclonal antibodies against SFTS virus Gn, M1-E5 and Ab10 clones, were prepared as described previously [39].

### Serial passage of SFTS virus

HeLa cells were infected with the SFTS virus SPL030 strain (the original strain) at an MOI of 2.5 and at 3 or 4 dpi culture supernatant containing proliferating virus was harvested and diluted (approximately 1:10 dilution) to infect fresh HeLa cells. This was repeated 50 times. To obtain a cloned virus, a limiting dilution with Vero cells was performed 3 times.

### Growth kinetics of SFTS virus in cell lines

Monolayers of Vero, HeLa, Huh-7, SK-N-SH cells were prepared and inoculated with viruses at an MOI of 0.025 for 1 h. The cells were washed 3 times and cultured for 3 days. Culture supernatant was harvested daily and titrated.

### Mouse models

For SFTS, *Ifnar*^-/-^ mice were used as described previously [39,59]. For HRTV, AG129 mice were used as described previously [35,48]. Inoculated mice were observed daily for body weight and signs of life. In chronological analyses, mice were anesthetized and the sampling of blood, liver, spleen, and brain was performed upon euthanasia. Aliquots of blood were used for complete blood counts using a Vetscan HM5 (Zoetis), biochemical analysis with sera by Vetscan VS2 (Zoetis), and cytokine/chemokine measurement with sera by a multiplex assay (Milliplex MAP Mouse Cytokine/Chemokine Magnetic Bead Panel, Merck). Total RNA was prepared from organs harvested using ISOGEN with a Spin Column (Nippon Gene Co., Ltd) and used as templates for viral copy numbers by quantitative real-time RT-PCR [60].

### Viral genome sequencing

Viral RNA was prepared from culture supernatants of inoculated cells using a High Pure Viral RNA Kit (Roche). RT-PCR was performed as described previously with purified viral RNA [60] and sequences were determined by Sanger sequencing.

### IFA

Infected monolayer cells were fixed with 10% formalin and exposed to ultraviolet light to inactivate virus. Cells were treated with PBS containing 0.2% Triton X-100 and incubated with rabbit anti-rNP serum (#75 [61]). After being washed with PBS, cells were further incubated with goat anti-rabbit IgG Alexa Fluor 488 (Invitrogen). After being washed, cells were observed under a fluorescence microscope (BZ-700, Keyence).

### Glycosylation status of SFTS virus GP

Infected Vero cell monolayers were harvested with a denaturing buffer followed by endoglycosidase H or Peptide-N-Glycosidase F treatment (New England Biolabs) according to the manufacturer’s protocols. SDS-PAGE was performed under a reduced condition. For western blotting, an anti-Gn human monoclonal antibody and anti-Gc rabbit polyclonal antibody were used as primary antibodies under the use of SuperSignal West Dura Extended Duration Substrate (Thermo Scientific).

### Intracellular staining flow cytometry

Infected cells were fixed with 10% formalin and Perm/Wash Buffer (BD Biosciences) and used for antibody staining and washing of cells. A mouse monoclonal antibody to the SFTS virus NP 9D3 [61] and goat anti-mouse IgG (H+L) Alexa Fluor 488 (Thermo Scientific) were used as primary and secondary antibodies, respectively, and stained cells were analyzed by FACSCalibur and Cell Quest Pro software (BD Biosciences). Fluorescein (Dojindo Molecular Technologies)-labeled monoclonal antibody (2D11) [61] was used for staining in some experiments.

### iVLP of the SFTS virus and C-type lectin usage

iVLP was prepared as described previously [27]. The pC030GP plasmid [38] was used to express the GP of the SFTS virus SPL030 original strain in transfected cells. To express GP with a mutation, PCR-based mutagenesis was performed with primers having intended sequences and amplified cDNA fragments were cloned into pCAGGS. Sequences of resultant plasmids were examined by Sanger sequencing. The infectivity of iVLP was examined in Vero cells by the detection of a fluorescence reporter protein by flow cytometry. iVLP was diluted so that the positivity of infected Vero cells was between 2% and 5% and Jurkat cells, which expressed either of the C-type lectins, were infected with iVLP at the same dilution as Vero cells. C-type lectin usage was shown as the following ratios: positivity (%) in Jurkat cells/positivity (%) in Vero cells.

### Recombinant viruses

Plasmids for SFTS virus reverse genetics (HB29 strain) were kindly provided by Dr. Benjamin Brennan [41]. Plasmids for the SFTS virus SPL030 original strain genome (pTV7OriL, pTV7OriM, pTV7OriS) were previously reported [27]. The open reading frames were replaced with sequences from passaged Hp50-4, SPL010, and SPL057 strains. To prepare plasmids with point mutations, PCR-based mutagenesis was performed with primers having intended sequences. Sequences of resultant plasmids were examined by Sanger sequencing. The production of recombinant SFTS viruses was performed according to Brennan’s method [41] and expanded using Vero cells. The production of recombinant HRTV was performed as described previously [35].

### Differentiated THP-1 cells

For differentiation to macrophage-like cells, THP-1 cells were cultured in the presence of 50 ng/mL PMA for 3 days in HydroCell plates (CellSeed Inc.). To measure cytokines produced from the cells, MILLEX Human Cytokine/Chemokine Magnetic Bead Panel (Millipore) and Luminex 200 instrument system were used.

### Statistical analyses

Welch’s *t*-test was used to determine the significance of differences between the means of two groups. Dunnett’s test was used to determine the significance of differences between one group and others. The log-rank test was used for survival curves. A p-value < 0.05 was considered to indicate statistical significance.

## Supporting information

S1 Fig Characteristics of passaged SFTS virus. Body weight changes of inoculated Ifnar-/- mice (Fig 1C) are shown per individual. ♰ indicates humane endpoint or death.
(PDF)

S2 Fig Growth kinetics of the original and Hp50-4 strains of the SFTS virus. Confluent monolayers of cell lines indicated were inoculated with the original or passaged Hp50-4 strain at a multiplicity of infection of 0.025 and cultured for 3 days. Culture supernatant harvested at indicated days were titrated in Vero cells. Shown are means and standard deviations (n = 3). Statistical comparison was performed between the means for each day (Welch’s t-test).
(PDF)

S3 Fig Chimeric M segment sequences and recombinant virus phenotypes (*in vitro*). (A) A panel of plasmids for M segment encoding chimeric sequences from the original and Hp50-4 are shown in focusing on mutations. (B) Recombinant viruses were produced with the plasmids in combination with plasmids for the original L and S segments to infect HeLa cells. Viral antigens in inoculated cells were stained with rabbit anti-NP serum. High magnification of white enclosures are shown in right.
(PDF)

S4 Fig Glycosylation status of the Gc of SFTS virus recombinants. Western blotting analysis of lysates of Vero cells infected with recOri or recOri(U123A) recombinants were performed with (+) or without (-) glycosidase treatment and anti-Gc antibody.
(PDF)

S5 Fig Murine C-type lectin usage of the SFTS virus strains. Jurkat cells expressing a control molecule or one of murine C-type lectins (SIGNR1, SIGNR3, and LSECtin) and Vero cells were inoculated with either recOri or recOri(U123A) strain. Ratios of positivity in Jurkat cells to positivity in Vero cells are shown. Data shown are means and standard deviations (n = 3). Statistical comparison was performed between control recOri(U123A) and others indicated (Dunnett’s test).
(PDF)

S6 Fig Inhibition of the C-type lectin-mediated infection by sugars. Jurkat cells expressing C-type lectins (DC-SIGN, DC-SIGNR, and LSECtin) were inoculated with iVLP in the presence of inhibitory sugars (Mannan or GlcNAcβ1-2Man) at indicated concentrations. Data shown are means (% of no inhibitor) and standard deviations (n = 3).
(PDF)

S7 Fig Infectivity of iVLPs with GPs having mutations in N-glycosylation motifs. Vero cells were inoculated with iVLPs at 1:5 and 1:25 dilutions and positivity (%) of reporter expression was measured with a flow cytometry. Positivity of uninoculated Vero cells was set as 0.92.
(PDF)

S8 Fig C-type lectin usage of iVLPs and virulence of the recOri(Δ1stNgly) strain. (A) Jurkat cells expressing a control molecule or one of human C-type lectins (DC-SIGN, DC-SIGNR, and LSECtin) and Vero cells were inoculated with iVLP carrying the original GP, Ori(U123A) GP, or GP lacking one N-glycosylation motif by asparagine-to-glutamine substitution (1st, 2nd, 3rd, or 5th). Ratios of reporter positivity in Jurkat cells to reporter positivity in Vero cells are shown. Data shown are means and standard deviations (n = 3). (B) Ifnar-/- mice were subcutaneously inoculated with 102 50% tissue culture infectious doses of indicated strains (four mice per group) and observed until 14 days post inoculation. Survival curves are shown.
(PDF)

S9 Fig C-type lectin usage and virulence of the SPL010- or SPL057-based recombinants. (A) SFTS virus recombinants were produced by reverse genetics, which had SPL010- or SPL057-strain backbone with (Δ1stNgly) or without (rec) lack of the 1st N-glycosylation of GP. Jurkat cells expressing control molecule or one of human C-type lectins (DC-SIGN, DC-SIGNR, and LSECtin) and Vero cells were inoculated at a multiplicity of infection of 0.025. Ratios of viral antigen positivity in Jurkat cells to viral antigen positivity in Vero cells are shown for SPL010 (left) and for SPL057 (right). Data shown are means and standard deviations (n = 3). (B) Ifnar-/- mice were subcutaneously inoculated with 102 50% tissue culture infectious doses of recombinant viruses (four mice per group) and observed until 14 days post inoculation. Survival curves are shown for SPL010 (left) and for SPL057 (right).
(PDF)

S10 Fig Effects of C-type lectin inhibitory antibodies on iVLP-Ori infection in PMA-treated THP-1 cells. Phorbol 12-myristate 13-acetate-treated THP-1 cells were infected with iVLP carrying the original GP in the presence of control mouse IgG or either (A) or combination (B) of antibody clones #120507 (DC-SIGN-specific), #120604 (DC-SIGNR-specific), and SOTO-1 (LSECtin-specific) (10 μg/mL each). Reporter expression were analyzed by flow cytometry and % of control (normal mouse IgG1) are shown. Data shown are means and standard deviations (n = 3).
(PDF)

S11 Fig Viral genome distribution and host response in a mouse model. Ifnar-/- mice were subcutaneously inoculated with 102 50% tissue culture infectious doses of recOri or recOri(U123A) and organ and blood sampling performed upon euthanasia at indicated days. RNA were extracted from organs (liver, spleen, kidney, and brain) to measure viral genome copy numbers. Sera were used to perform biochemical test (albumin, blood urea nitrogen, glucose, and globulin) and ELISA (IL-1β, IL-10, IL-13, IP-10, MCP-1, and MIP-1α). Blue and orange bars are for recOri- and recOri(U123A)-infected mice, respectively. Black bars are data for mice injected with control media (6 days post injection). Three mice per group were used at each sampling points. Data shown are means and standard deviations (n = 3).
(PDF)

S12 Fig Infectivity of SFTS virus replicating *in vivo* Sera harvested from Ifnar-/- mice were used for titration in Vero cells and for inoculation of Jurkat cells expressing C-type lectins and control Jurkat cells. Titers are shown in parentheses (TCID50, log10/mL). Infectivity in Jurkat cells were measured as positivity (%) in intracellular staining flow cytometry with fluorescein-labeled anti-SFTS virus NP monoclonal antibody. recOri and recOri(U123A) viruses prepared in vitro were also used as control viruses.
(PDF)

S13 Fig Alignment of amino acid sequences of the GP of SFTS virus and its related viruses. Amino acid sequences of GP of indicated viruses were aligned using GENETYX software. Accession numbers of sequences used are shown in parentheses. Regions including five N-linked glycosylation motifs of SFTS virus GP are shown. Red bolds are N-linked glycosylation motifs. GTV, Guertu virus; HRTV, Heartland virus; HIGV, Hunter Island group virus. Stars indicate conserved amino acids among the aligned sequences.
(PDF)

S14 Fig Body weight of AG129 mice inoculated with the Heartland virus. Mean body weight of inoculated AG129 mice (Fig 8) are shown.
(PDF)

S1 Data Raw data of main figures. (XLSX)

S2 Data Raw data of supporting information figures. (XLSX)
