# Supplementary material for: N-glycosylation of viral glycoprotein is a novel determinant for the tropism and virulence of highly pathogenic tick-borne bunyaviruses
Source: PLoS Pathog. 2024 Jul 15;20(7):e1012348. doi: 10.1371/journal.ppat.1012348 (PMC11271937; doi:10.1371/journal.ppat.1012348)
Supplement: S1 Fig — Body weight changes of inoculated Ifnar-/- mice (Fig 1C) are shown per individual. ♰ indicates humane endpoint or death. (PDF) [file ppat.1012348.s001.pdf]

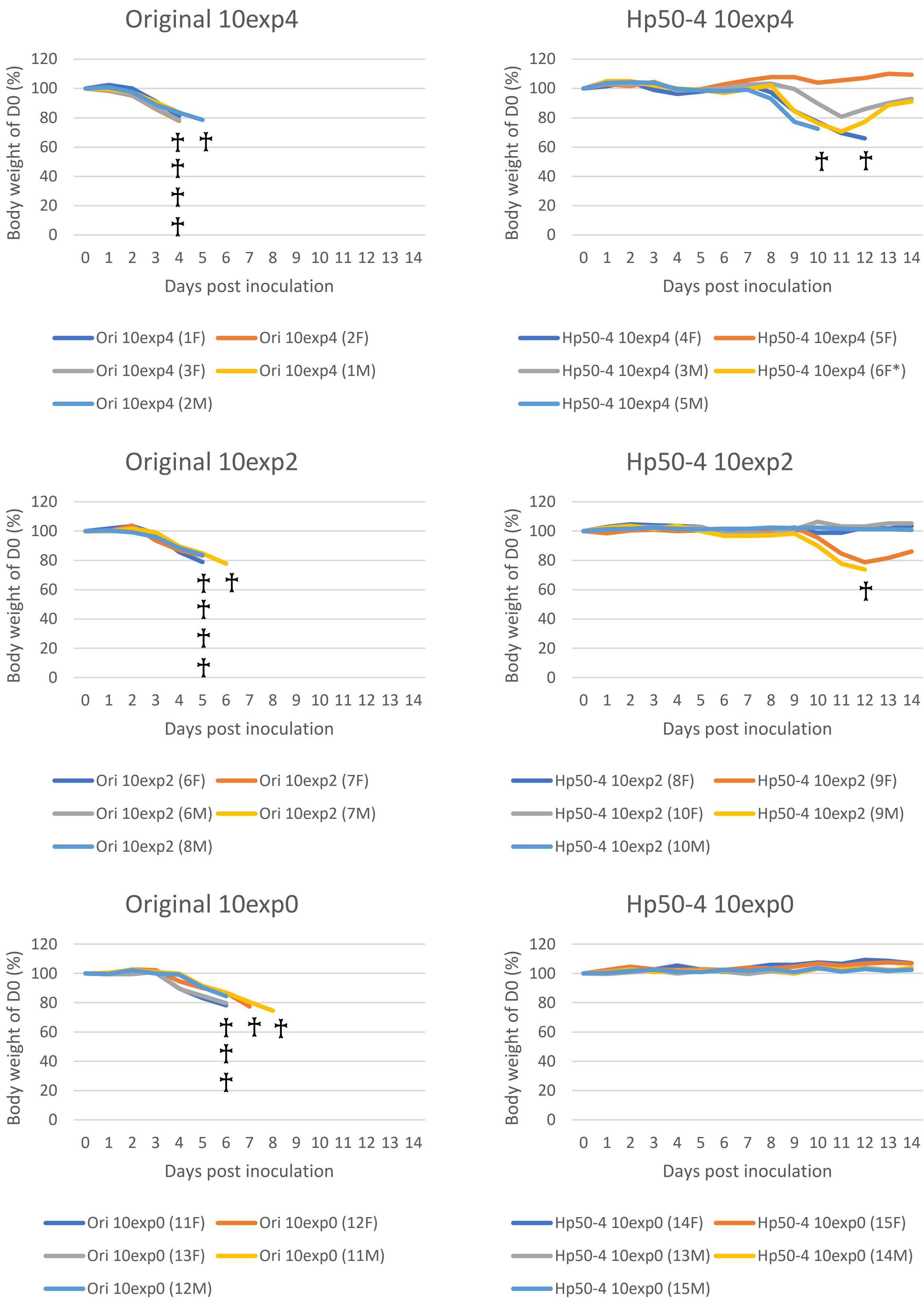

S1 Fig: Characteristics of passaged SFTS virus

Body weight changes of inoculated Ifnar$^{-/-}$ mice (Fig 1C) are shown per individual. † indicates humane endpoint or death.
