# Supplementary material for: N-glycosylation of viral glycoprotein is a novel determinant for the tropism and virulence of highly pathogenic tick-borne bunyaviruses
Source: PLoS Pathog. 2024 Jul 15;20(7):e1012348. doi: 10.1371/journal.ppat.1012348 (PMC11271937; doi:10.1371/journal.ppat.1012348)
Supplement: S2 Fig — Confluent monolayers of cell lines indicated were inoculated with the original or passaged Hp50-4 strain at a multiplicity of infection of 0.025 and cultured for 3 days. Culture supernatant harvested at indicated days were titrated in Vero cells. Shown are means and standard deviations (n = 3). Statistical comparison was performed between the means for each day (Welch’s t-test). (PDF) [file ppat.1012348.s002.pdf]

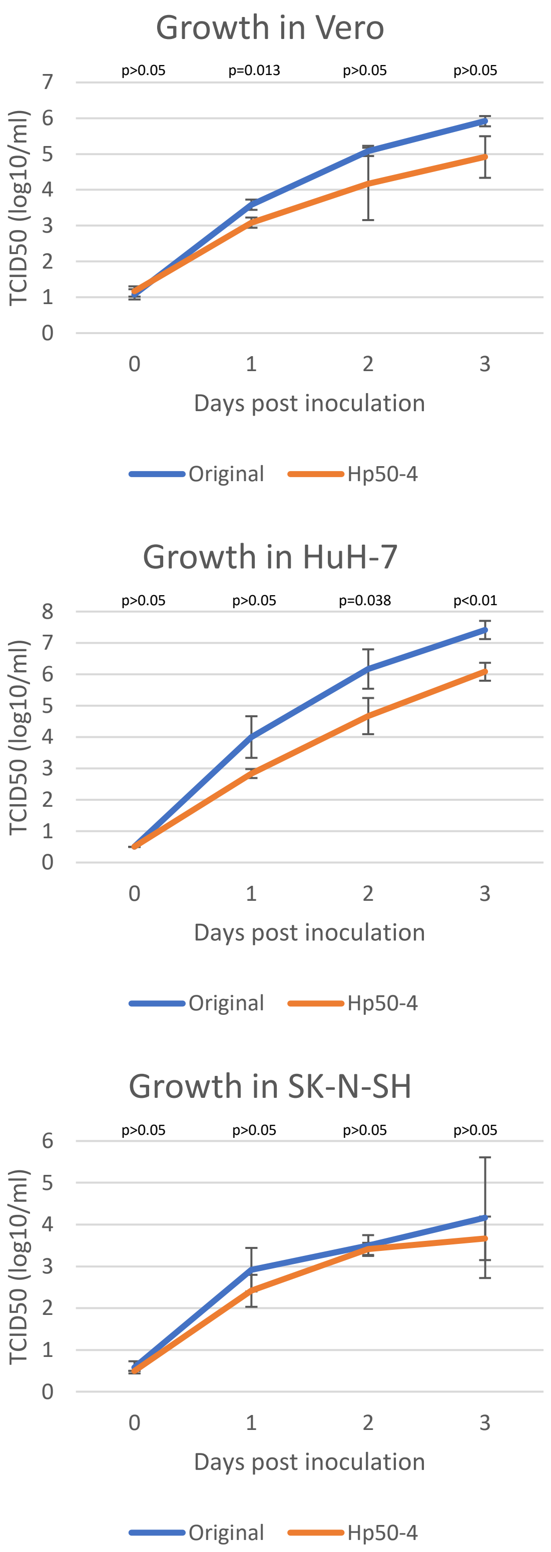

S2 Fig: Growth kinetics of the original and Hp50-4 strains of the SFTS virus

Confluent monolayers of cell lines indicated were inoculated with the original or passaged Hp50-4 strain at a multiplicity of infection of 0.025 and cultured for 3 days. Culture supernatant harvested at indicated days were titrated in Vero cells. Shown are means and standard deviations (n=3). Statistical comparison was performed between the means for each day (Welch's *t*-test).
