# Supplementary material for: N-glycosylation of viral glycoprotein is a novel determinant for the tropism and virulence of highly pathogenic tick-borne bunyaviruses
Source: PLoS Pathog. 2024 Jul 15;20(7):e1012348. doi: 10.1371/journal.ppat.1012348 (PMC11271937; doi:10.1371/journal.ppat.1012348)
Supplement: S3 Fig — (A) A panel of plasmids for M segment encoding chimeric sequences from the original and Hp50-4 are shown in focusing on mutations. (B) Recombinant viruses were produced with the plasmids in combination with plasmids for the original L and S segments to infect HeLa cells. Viral antigens in inoculated cells were stained with rabbit anti-NP serum. High magnification of white enclosures are shown in right. (PDF) [file ppat.1012348.s003.pdf]

A

Position in the M segment	123	500	922	1720
recOri	U	G	G	C
rHHHH	A	A	A	U
rOOHH	U	G	A	U
rHHOO	A	A	G	C
$recL_{Ori}M_{Hp}$(A123U)	U	A	A	U
recOri(U123A)	A	G	G	C
rOHOO	U	A	G	C
rHOHH	A	G	A	U

B

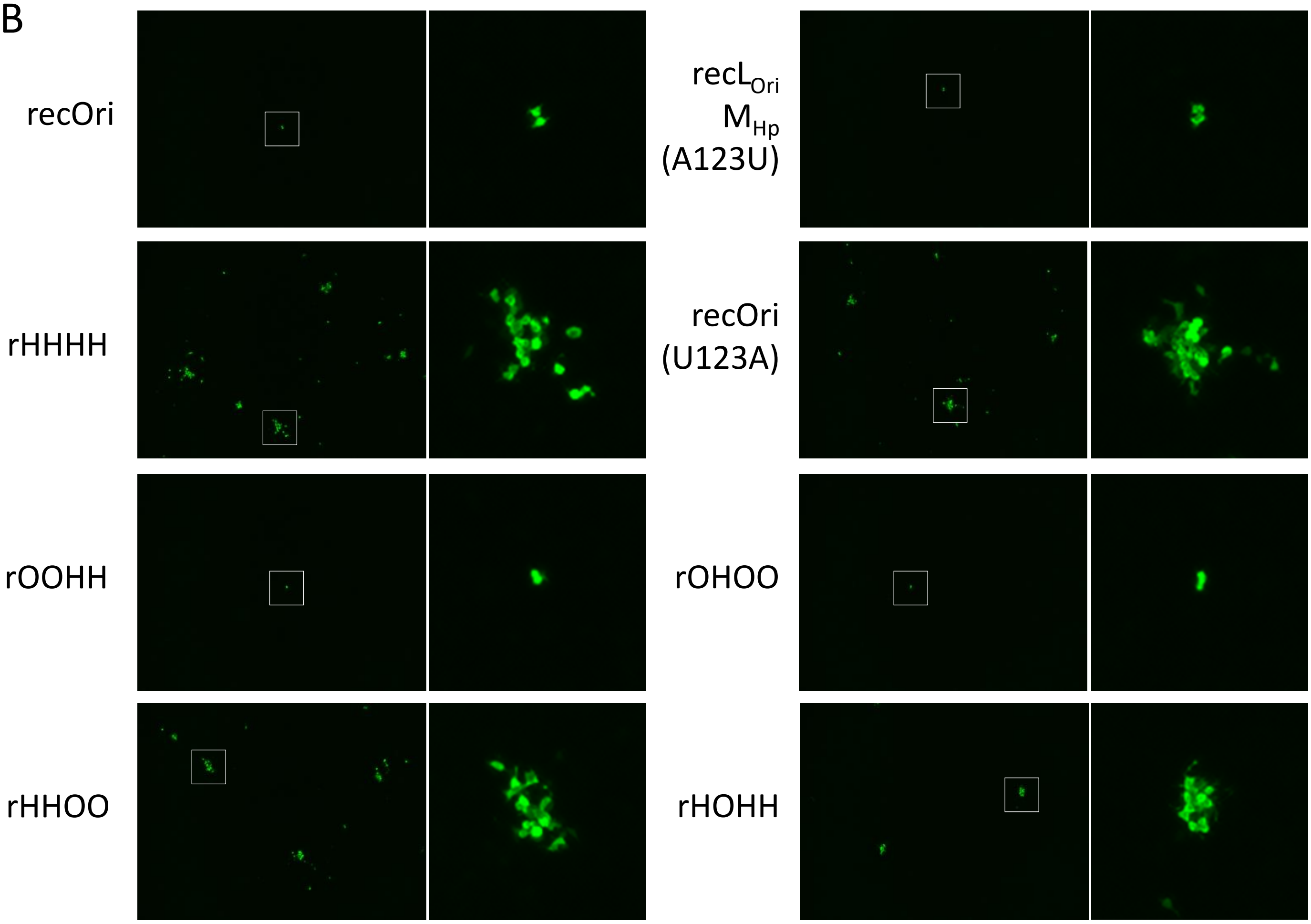

S3 Fig: Chimeric M segment sequences and recombinant virus phenotypes (in vitro)

(A) A panel of plasmids for M segment encoding chimeric sequences from the original and Hp50-4 are shown in focusing on mutations. (B) Recombinant viruses were produced with the plasmids in combination with plasmids for the original L and S segments to infect HeLa cells. Viral antigens in inoculated cells were stained with rabbit anti-NP serum. High magnification of white enclosures are shown in right.
