# Supplementary material for: N-glycosylation of viral glycoprotein is a novel determinant for the tropism and virulence of highly pathogenic tick-borne bunyaviruses
Source: PLoS Pathog. 2024 Jul 15;20(7):e1012348. doi: 10.1371/journal.ppat.1012348 (PMC11271937; doi:10.1371/journal.ppat.1012348)
Supplement: S4 Fig — Western blotting analysis of lysates of Vero cells infected with recOri or recOri(U123A) recombinants were performed with (+) or without (-) glycosidase treatment and anti-Gc antibody. (PDF) [file ppat.1012348.s004.pdf]

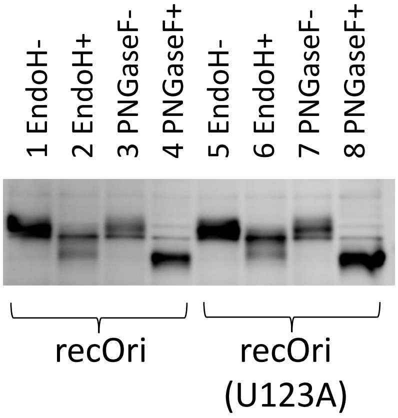

S4 Fig: Glycosylation status of the Gc of SFTS virus recombinants

Western blotting analysis of lysates of Vero cells infected with recOri or recOri(U123A) recombinants were performed with (+) or without (-) glycosidase treatment and anti-Gc antibody.
