# Supplementary material for: N-glycosylation of viral glycoprotein is a novel determinant for the tropism and virulence of highly pathogenic tick-borne bunyaviruses
Source: PLoS Pathog. 2024 Jul 15;20(7):e1012348. doi: 10.1371/journal.ppat.1012348 (PMC11271937; doi:10.1371/journal.ppat.1012348)
Supplement: S5 Fig — Jurkat cells expressing a control molecule or one of murine C-type lectins (SIGNR1, SIGNR3, and LSECtin) and Vero cells were inoculated with either recOri or recOri(U123A) strain. Ratios of positivity in Jurkat cells to positivity in Vero cells are shown. Data shown are means and standard deviations (n = 3). Statistical comparison was performed between control recOri(U123A) and others indicated (Dunnett’s test). (PDF) [file ppat.1012348.s005.pdf]

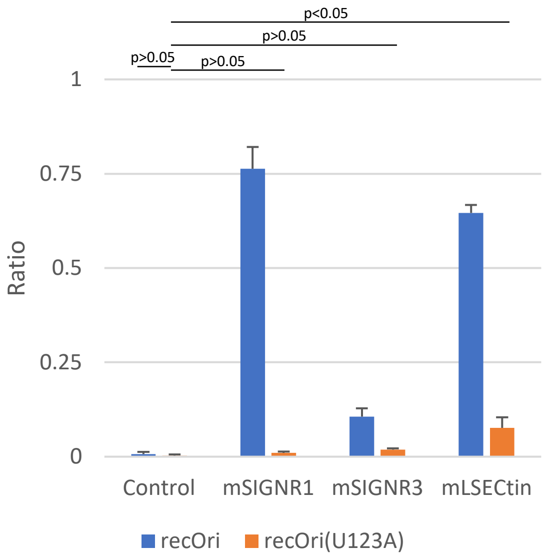

S5 Fig: Murine C-type lectin usage of the SFTS virus strains

Jurkat cells expressing a control molecule or one of murine C-type lectins (SIGNR1, SIGNR3, and LSECtin) and Vero cells were inoculated with either recOri or recOri(U123A) strain. Ratios of positivity in Jurkat cells to positivity in Vero cells are shown. Data shown are means and standard deviations (n=3). Statistical comparison was performed between control recOri(U123A) and others indicated (Dunnett's test).
