# Supplementary material for: N-glycosylation of viral glycoprotein is a novel determinant for the tropism and virulence of highly pathogenic tick-borne bunyaviruses
Source: PLoS Pathog. 2024 Jul 15;20(7):e1012348. doi: 10.1371/journal.ppat.1012348 (PMC11271937; doi:10.1371/journal.ppat.1012348)
Supplement: S6 Fig — Jurkat cells expressing C-type lectins (DC-SIGN, DC-SIGNR, and LSECtin) were inoculated with iVLP in the presence of inhibitory sugars (Mannan or GlcNAcβ1-2Man) at indicated concentrations. Data shown are means (% of no inhibitor) and standard deviations (n = 3). (PDF) [file ppat.1012348.s006.pdf]

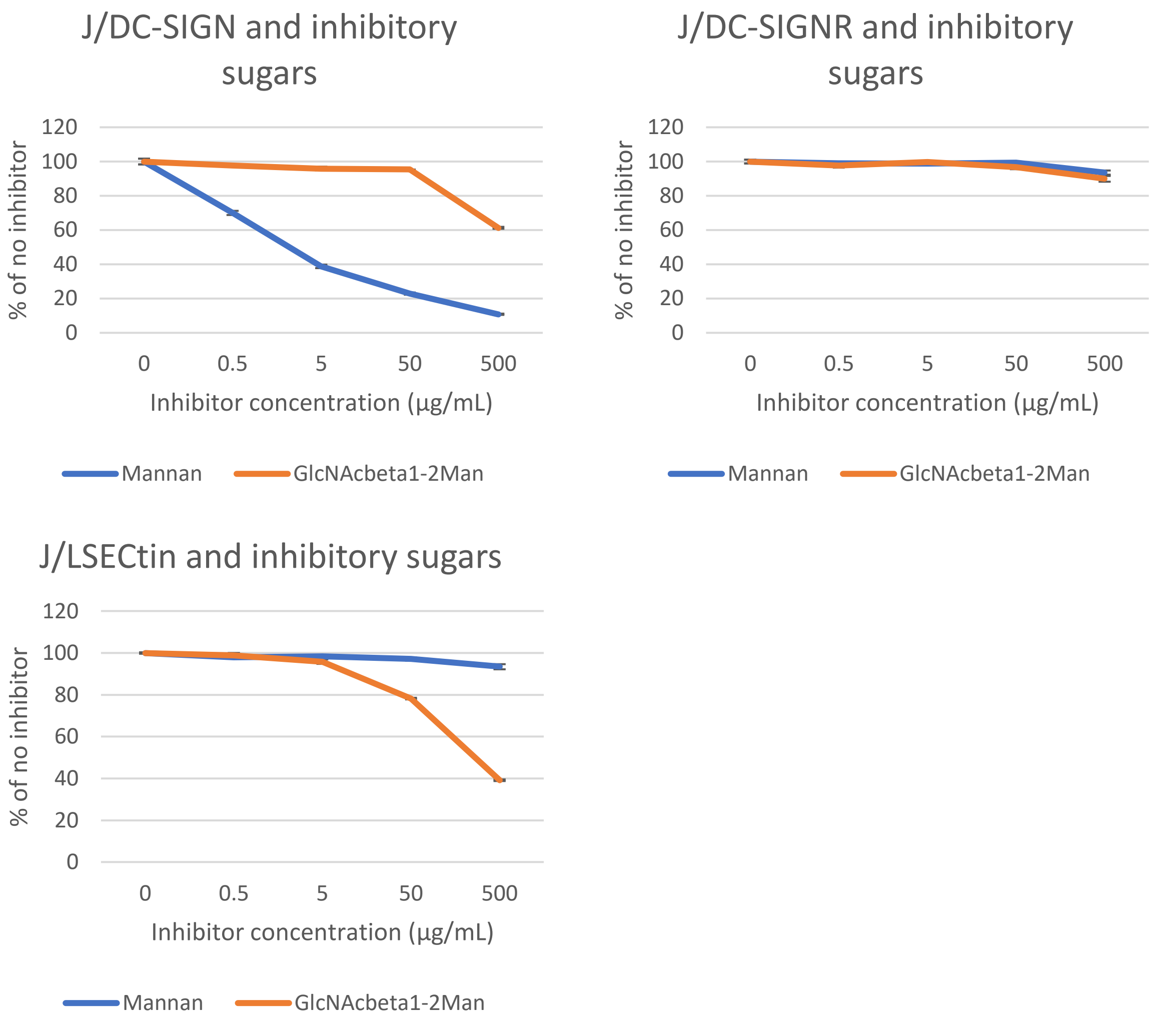

S6 Fig: Inhibition of the C-type lectin-mediated infection by sugars

Jurkat cells expressing C-type lectins (DC-SIGN, DC-SIGNR, and LSECtin) were inoculated with iVLP in the presence of inhibitory sugars (Mannan or GlcNAcβ1-2Man) at indicated concentrations. Data shown are means (% of no inhibitor) and standard deviations (n=3).
