# Supplementary material for: N-glycosylation of viral glycoprotein is a novel determinant for the tropism and virulence of highly pathogenic tick-borne bunyaviruses
Source: PLoS Pathog. 2024 Jul 15;20(7):e1012348. doi: 10.1371/journal.ppat.1012348 (PMC11271937; doi:10.1371/journal.ppat.1012348)
Supplement: S7 Fig — Vero cells were inoculated with iVLPs at 1:5 and 1:25 dilutions and positivity (%) of reporter expression was measured with a flow cytometry. Positivity of uninoculated Vero cells was set as 0.92. (PDF) [file ppat.1012348.s007.pdf]

	1:5 dilution	1:25 dilution
GP(Ori)	Not done	86.53
GP(Ori(U123A))	17.96	4.52
GP(Δ1stNgly)	84.72	56.90
GP(Δ2ndNgly)	78.63	51.94
GP(Δ3rdNgly)	67.12	34.59
GP(Δ4thNgly)	1.28	1.00
GP(Δ5thNgly)	Not done	86.58

S7 Fig: Infectivity of iVLPs with GPs having mutations in N-glycosylation motifs
Vero cells were inoculated with iVLPs at 1:5 and 1:25 dilutions and positivity (%) of reporter expression was measured with a flow cytometry. Positivity of uninoculated Vero cells was set as 0.92%.
