# Supplementary material for: N-glycosylation of viral glycoprotein is a novel determinant for the tropism and virulence of highly pathogenic tick-borne bunyaviruses
Source: PLoS Pathog. 2024 Jul 15;20(7):e1012348. doi: 10.1371/journal.ppat.1012348 (PMC11271937; doi:10.1371/journal.ppat.1012348)
Supplement: S8 Fig — (A) Jurkat cells expressing a control molecule or one of human C-type lectins (DC-SIGN, DC-SIGNR, and LSECtin) and Vero cells were inoculated with iVLP carrying the original GP, Ori(U123A) GP, or GP lacking one N-glycosylation motif by asparagine-to-glutamine substitution (1st, 2nd, 3rd, or 5th). Ratios of reporter positivity in Jurkat cells to reporter positivity in Vero cells are shown. Data shown are means and standard deviations (n = 3). (B) Ifnar-/- mice were subcutaneously inoculated with 102 50% tissue culture infectious doses of indicated strains (four mice per group) and observed until 14 days post inoculation. Survival curves are shown. (PDF) [file ppat.1012348.s008.pdf]

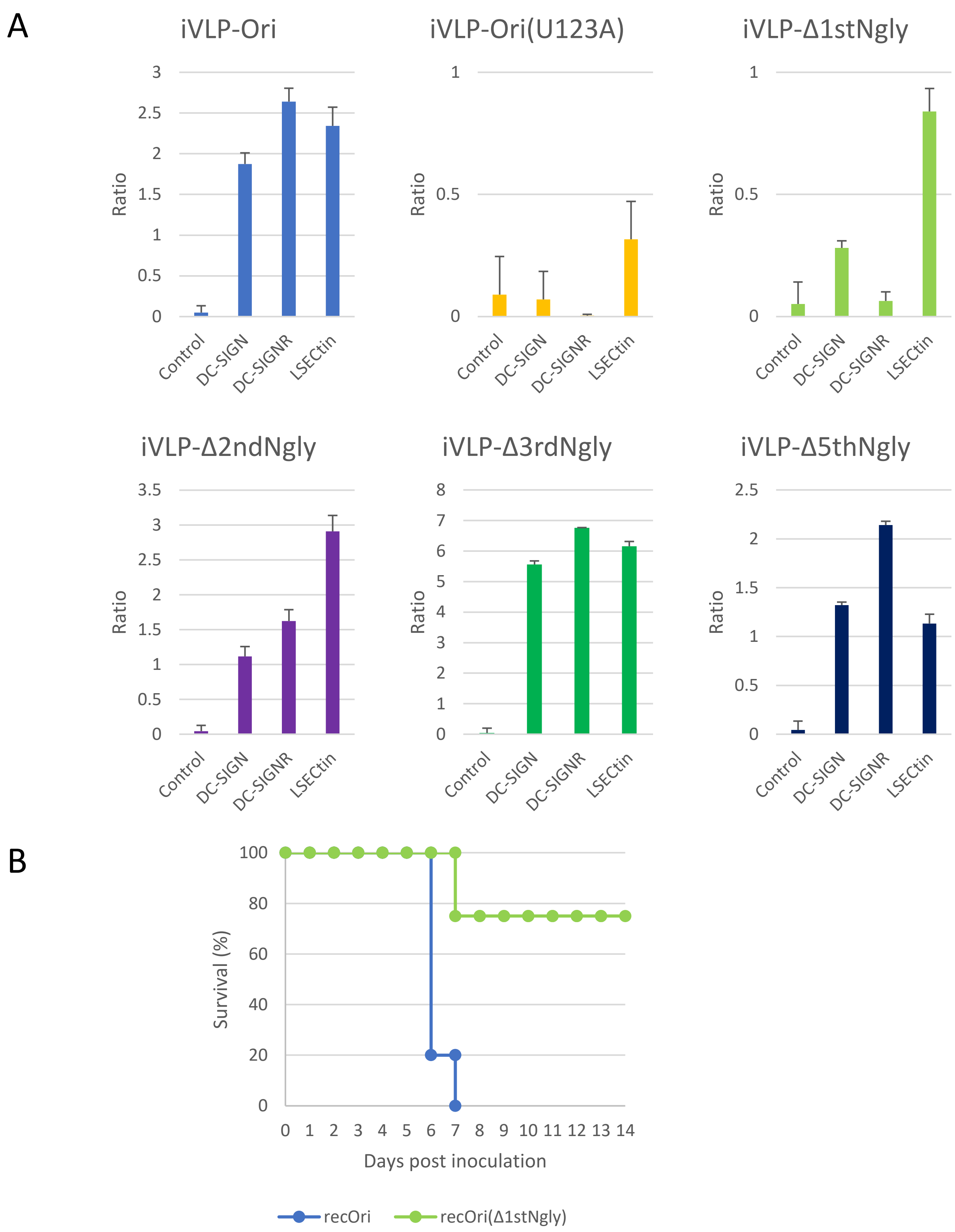

S8 Fig: C-type lectin usage of iVLPs and virulence of the recOri(Δ1stNgly) strain

(A) Jurkat cells expressing a control molecule or one of human C-type lectins (DC-SIGN, DC-SIGNR, and LSECtin) and Vero cells were inoculated with iVLP carrying the original GP, Ori(U123A) GP, or GP lacking one N-glycosylation motif by asparagine-to-glutamine substitution (1st, 2nd, 3rd, or 5th). Ratios of reporter positivity in Jurkat cells to reporter positivity in Vero cells are shown. Data shown are means and standard deviations (n=3). (B) Ifnar$^{-/-}$ mice were subcutaneously inoculated with 10^2 50% tissue culture infectious doses of indicated strains (four mice per group) and observed until 14 days post inoculation. Survival curves are shown.
