# Supplementary material for: N-glycosylation of viral glycoprotein is a novel determinant for the tropism and virulence of highly pathogenic tick-borne bunyaviruses
Source: PLoS Pathog. 2024 Jul 15;20(7):e1012348. doi: 10.1371/journal.ppat.1012348 (PMC11271937; doi:10.1371/journal.ppat.1012348)
Supplement: S9 Fig — (A) SFTS virus recombinants were produced by reverse genetics, which had SPL010- or SPL057-strain backbone with (Δ1stNgly) or without (rec) lack of the 1st N-glycosylation of GP. Jurkat cells expressing control molecule or one of human C-type lectins (DC-SIGN, DC-SIGNR, and LSECtin) and Vero cells were inoculated at a multiplicity of infection of 0.025. Ratios of viral antigen positivity in Jurkat cells to viral antigen positivity in Vero cells are shown for SPL010 (left) and for SPL057 (right). Data shown are means and standard deviations (n = 3). (B) Ifnar-/- mice were subcutaneously inoculated with 102 50% tissue culture infectious doses of recombinant viruses (four mice per group) and observed until 14 days post inoculation. Survival curves are shown for SPL010 (left) and for SPL057 (right). (PDF) [file ppat.1012348.s009.pdf]

A

B

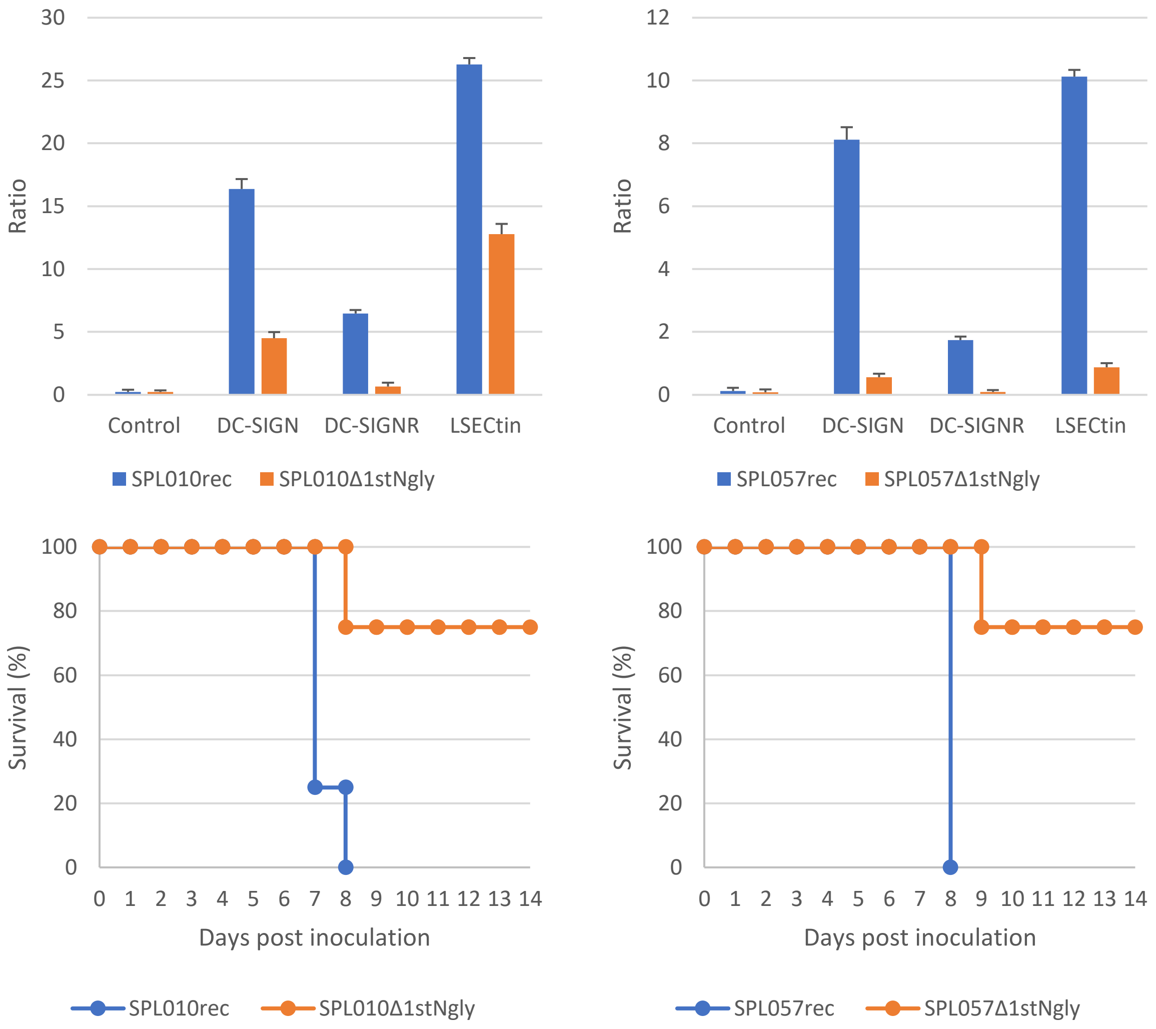

S9 Fig: C-type lectin usage and virulence of the SPL010- or SPL057-based recombinants
(A) SFTS virus recombinants were produced by reverse genetics, which had SPL010- or SPL057-strain backbone with (Δ1stNgly) or without (rec) lack of the 1st N-glycosylation of GP. Jurkat cells expressing control molecule or one of human C-type lectins (DC-SIGN, DC-SIGNR, and LSECtin) and Vero cells were inoculated at a multiplicity of infection of 0.025. Ratios of viral antigen positivity in Jurkat cells to viral antigen positivity in Vero cells are shown for SPL010 (left) and for SPL057 (right). Data shown are means and standard deviations (n=3). (B) Ifnar$^{-/-}$ mice were subcutaneously inoculated with 10^2 50% tissue culture infectious doses of recombinant viruses (four mice per group) and observed until 14 days post inoculation. Survival curves are shown for SPL010 (left) and for SPL057 (right).
