# Supplementary material for: N-glycosylation of viral glycoprotein is a novel determinant for the tropism and virulence of highly pathogenic tick-borne bunyaviruses
Source: PLoS Pathog. 2024 Jul 15;20(7):e1012348. doi: 10.1371/journal.ppat.1012348 (PMC11271937; doi:10.1371/journal.ppat.1012348)
Supplement: S10 Fig — Phorbol 12-myristate 13-acetate-treated THP-1 cells were infected with iVLP carrying the original GP in the presence of control mouse IgG or either (A) or combination (B) of antibody clones #120507 (DC-SIGN-specific), #120604 (DC-SIGNR-specific), and SOTO-1 (LSECtin-specific) (10 μg/mL each). Reporter expression were analyzed by flow cytometry and % of control (normal mouse IgG1) are shown. Data shown are means and standard deviations (n = 3). (PDF) [file ppat.1012348.s010.pdf]

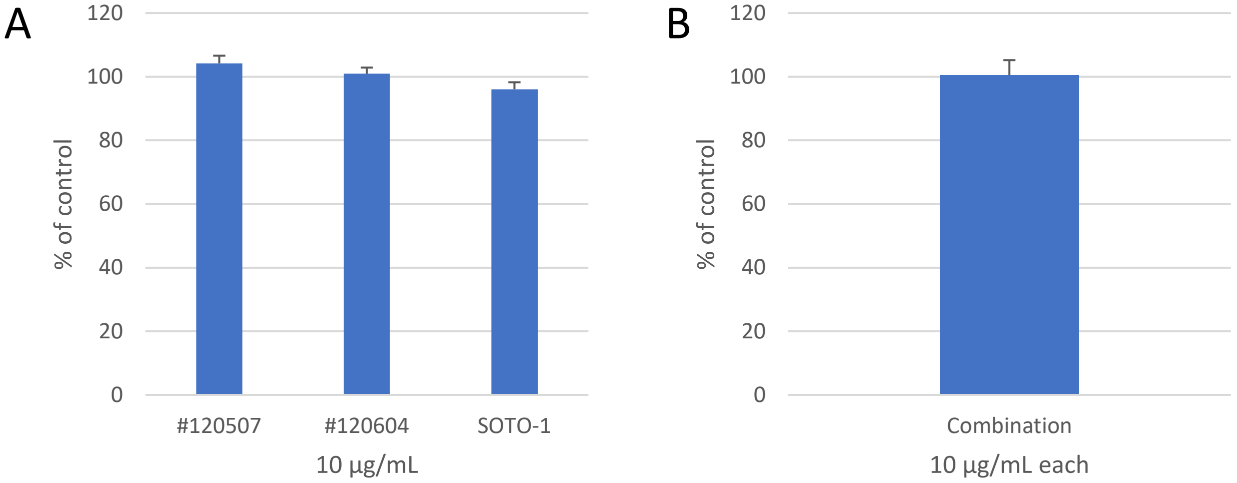

S10 Fig: Effects of C-type lectin inhibitory antibodies on iVLP-Ori infection in PMA-treated THP-1 cells

Phorbol 12-myristate 13-acetate-treated THP-1 cells were infected with iVLP carrying the original GP in the presence of control mouse IgG or either (A) or combination (B) of antibody clones #120507 (DC-SIGN-specific), #120604 (DC-SIGNR-specific), and SOTO-1 (LSECtin-specific) (10 µg/mL each). Reporter expression were analyzed by flow cytometry and % of control (normal mouse IgG1) are shown. Data shown are means and standard deviations (n=3).
