# Supplementary material for: N-glycosylation of viral glycoprotein is a novel determinant for the tropism and virulence of highly pathogenic tick-borne bunyaviruses
Source: PLoS Pathog. 2024 Jul 15;20(7):e1012348. doi: 10.1371/journal.ppat.1012348 (PMC11271937; doi:10.1371/journal.ppat.1012348)
Supplement: S11 Fig — Ifnar-/- mice were subcutaneously inoculated with 102 50% tissue culture infectious doses of recOri or recOri(U123A) and organ and blood sampling performed upon euthanasia at indicated days. RNA were extracted from organs (liver, spleen, kidney, and brain) to measure viral genome copy numbers. Sera were used to perform biochemical test (albumin, blood urea nitrogen, glucose, and globulin) and ELISA (IL-1β, IL-10, IL-13, IP-10, MCP-1, and MIP-1α). Blue and orange bars are for recOri- and recOri(U123A)-infected mice, respectively. Black bars are data for mice injected with control media (6 days post injection). Three mice per group were used at each sampling points. Data shown are means and standard deviations (n = 3). (PDF) [file ppat.1012348.s011.pdf]

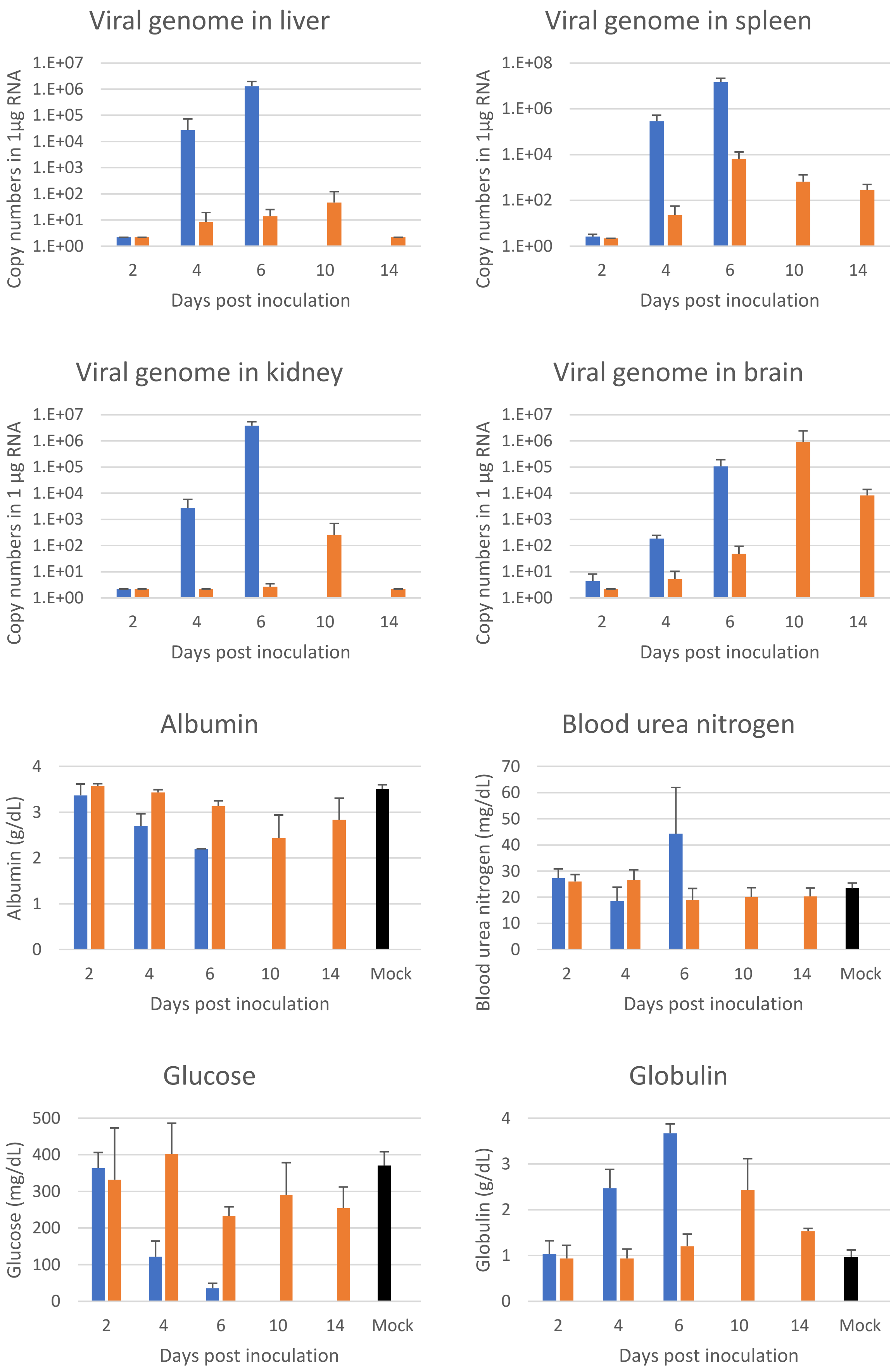

S11 Fig: Viral genome distribution and host response in a mouse model

(Continues to next sheet)

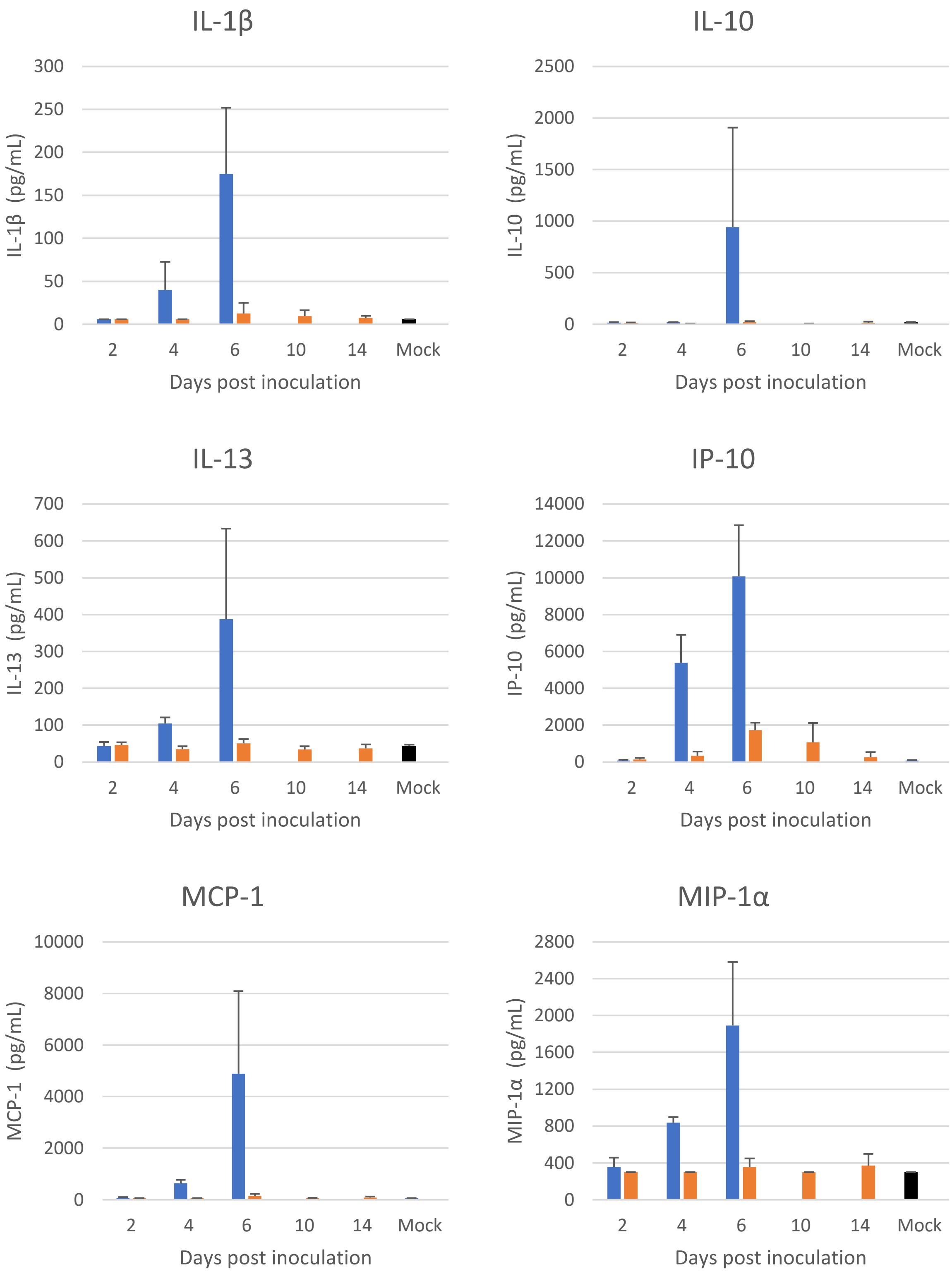

S11 Fig: Viral genome distribution and host response in a mouse model

(Continues from previous sheet)

Ifnar$^{-/-}$ mice were subcutaneously inoculated with 10^2 50% tissue culture infectious doses of recOri or recOri(U123A) and organ and blood sampling performed upon euthanasia at indicated days. RNA were extracted from organs (liver, spleen, kidney, and brain) to measure viral genome copy numbers. Sera were used to perform biochemical test (albumin, blood urea nitrogen, glucose, and globulin) and ELISA (IL-1β, IL-10, IL-13, IP-10, MCP-1, and MIP-1α). Blue and orange bars are for recOri- and recOri(U123A)-infected mice, respectively. Black bars are data for mice injected with control media (6 days post injection). Three mice per group were used at each sampling points. Data shown are means and standard deviations (n=3).
