# Supplementary material for: N-glycosylation of viral glycoprotein is a novel determinant for the tropism and virulence of highly pathogenic tick-borne bunyaviruses
Source: PLoS Pathog. 2024 Jul 15;20(7):e1012348. doi: 10.1371/journal.ppat.1012348 (PMC11271937; doi:10.1371/journal.ppat.1012348)
Supplement: S12 Fig — Titers are shown in parentheses (TCID50, log10/mL). Infectivity in Jurkat cells were measured as positivity (%) in intracellular staining flow cytometry with fluorescein-labeled anti-SFTS virus NP monoclonal antibody. recOri and recOri(U123A) viruses prepared in vitro were also used as control viruses. (PDF) [file ppat.1012348.s012.pdf]

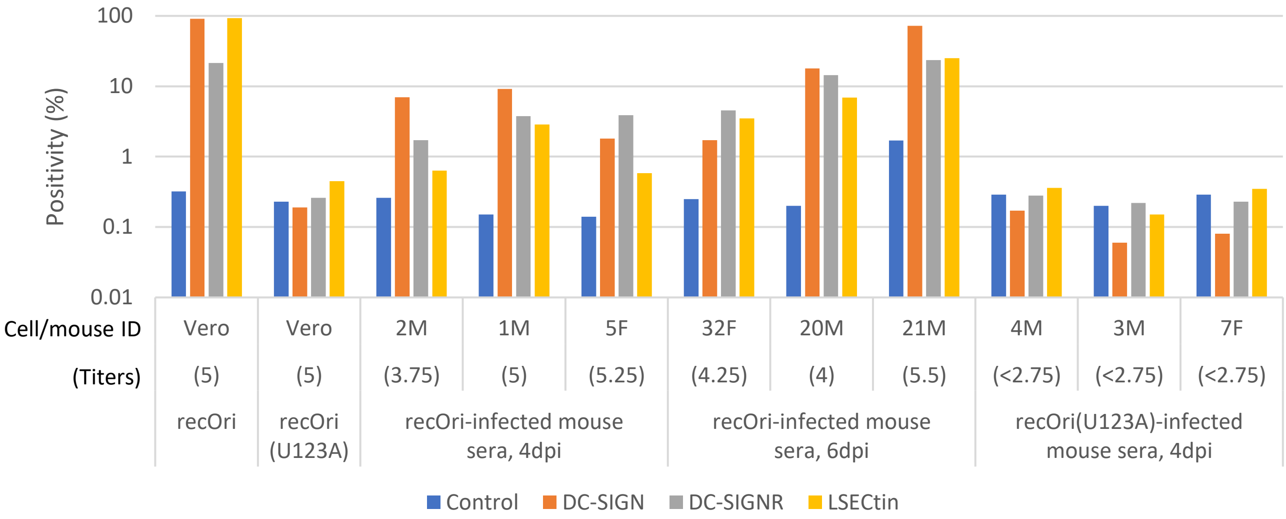

S12 Fig: Infectivity of SFTS virus replicating *in vivo*

Sera harvested from *Ifnar*$^{-/-}$ mice were used for titration in Vero cells and for inoculation of Jurkat cells expressing C-type lectins and control Jurkat cells. Titers are shown in parentheses (TCID50, log10/mL). Infectivity in Jurkat cells were measured as positivity (%) in intracellular staining flow cytometry with fluorescein-labeled anti-SFTS virus NP monoclonal antibody. recOri and recOri(U123A) viruses prepared in vitro were also used as control viruses.
