# Supplementary material for: N-glycosylation of viral glycoprotein is a novel determinant for the tropism and virulence of highly pathogenic tick-borne bunyaviruses
Source: PLoS Pathog. 2024 Jul 15;20(7):e1012348. doi: 10.1371/journal.ppat.1012348 (PMC11271937; doi:10.1371/journal.ppat.1012348)
Supplement: S13 Fig — Amino acid sequences of GP of indicated viruses were aligned using GENETYX software. Accession numbers of sequences used are shown in parentheses. Regions including five N-linked glycosylation motifs of SFTS virus GP are shown. Red bolds are N-linked glycosylation motifs. GTV, Guertu virus; HRTV, Heartland virus; HIGV, Hunter Island group virus. Stars indicate conserved amino acids among the aligned sequences. (PDF) [file ppat.1012348.s013.pdf]

```
SFTS virus GP (AB817992)    1:---MMKVIWFSSLICLVIQCSGDTSPIICAGPIHSNKSASIPHLLGYSEKICQIDRLIHV 57
GTV GP (NC_043609)          1:----MDFLWL-TLM-FLFWSARAEGPIICEGLTHSNKSAAIPHLLGYSEKMCQIDRLIHV 54
GTV GP (KT328592)           1:----MDFLWL-TLM-FLFWSARAEGPIICEGLTHSNKSAAIPHLLGYSEKMCQIDRLIHV 54
GTV GP (MH688509)           1:----MDFLWL-TLM-FLFWSARAEGPIICEGLTHSNKSAAIPHLLGYSEKMCQIDRLIHV 54
HRTV GP (MZ617375)          1:--------LTLCPSELSAWGSPGD-PIVCGVRTETNKSIQIEWKEGRSEKLCQIDRLGHV 51
HRTV GP (MZ617372)          1:MIVPIVLFLTLCPSELSAWGSPGD-PIVCGVRTETNKSIQIEWKEGRSEKLCQIDRLGHV 59
HRTV GP (MZ617369)          1:-----VLFLTLCPSGLSAWGSPGD-PIVCGVRTETNKSIQIEWKEGRSEKLCQIDRLGHV 54
HRTV GP (KJ740147)          1:MIVPIVLFLTLCPSELSAWGSPGD-PIVCGVRTETNKSIQIEWKEGRSEKLCQIDRLGHV 59
HRTV GP (NC_024494)         1:MIVPIVLFLTLCPSELSAWGSPGD-PIVCGVRTETNKSIQIEWKEGRSEKLCQIDRLGHV 59
HRTV GP (JX005845)          1:MIAPVVLFFTLCPSQLSAWGSPGD-PIVCGVRTETNKSIQIEWKEGRSEKLCQIDRLGHV 59
HRTV GP (JX005844)          1:MIVPIVLFLTLCPSELSAWGSPGD-PIVCGVRTETNKSIQIEWKEGRSEKLCQIDRLGHV 59
HIGV GP (NC_027715)         1:--------MEVVLLLLMLPGVSLGRNIICRADSRSEKNATVENHHGFSERYCQLDRLEHV 52
HIGV GP (KM198926)          1:--------MEVVLLLLMLPGVSLGRNIICRADSRSEKNATVENHHGFSERYCQLDRLEHV 52
HIGV GP (KF848981)          1:--------MEVVLLLLMLPGVSLGRNIICGADSRSEKNATVENHHGFSERYCQLDRLEHV 52
                                                        * *       *        * **  ** *** **

SFTS virus GP (AB817992)   58:SSWLRNHSQFQGYVGQRGGRSQVSYFPAENSYS-RWSGLLSPCDADWLGMLVVKKAKGSD 116
GTV GP (NC_043609)         55:SSWLRNHTQFEGFVGHRGGRSQVRYFPAENSYS-KWAGLLSPCDADWLGLLVVKKASQSD 113
GTV GP (KT328592)          55:SSWLRNHTQFEGFVGHRGGRSQVRYFPAENSYS-KWAGLLSPCDADWLGLLVVKKASQSD 113
GTV GP (MH688509)          55:SSWLRNHTQFEGFVGHRGGRSQVRYFPAENSYS-KWAGLLSPCDADWLGLLVVKKASQSD 113
HRTV GP (MZ617375)         52:TSWLRNHSSFQGLIGQVKGRPSVSYFPEGASYP-RWSGLLSPCDAEWLGLIAVSKAGDTD 110
HRTV GP (MZ617372)         60:TSWLRNHSSFQGLIGQVKGRPSVSYFPEGASYP-RWSGLLSPCDAEWLGLIAVSKAGDTD 118
HRTV GP (MZ617369)         55:TSWLRNHSSFQGLIGQVKGRPSVSYFPEGASYP-RWSGLLSPCDADWLGLIAVSKAGDTD 113
HRTV GP (KJ740147)         60:TSWLRNHSSFQGLVGQVKGRPSVSYFPEGASYP-RWSGLLSPCDAEWLGMIAVSKAGDTD 118
HRTV GP (NC_024494)        60:TSWLRNHSSFQGLIGQVKGRPSVSYFPEGASYP-RWSGLLSPCDAEWLGLIAVSKAGDTD 118
HRTV GP (JX005845)         60:TSWLRNHSSFQGLIGQVKGRPSVSYFPEGASYP-RWSGLLSPCDAEWLGLIAVSKAGDTD 118
HRTV GP (JX005844)         60:TSWLRNHSSFQGLIGQVKGRPSVSYFPEGASYP-RWSGLLSPCDAEWLGLIAVSKAGDTD 118
HIGV GP (NC_027715)        53:TSWMRNNSAFTGKVGMYQGRDKVEYFPAEENFQWKWPGLLSPCDADWMGIIRVVPAPKGV 112
HIGV GP (KM198926)         53:TSWMRNNSAFTGKVGMYQGRDKVEYFPAEENFQWKWPGLLSPCDADWMGIIRVVPAPKGV 112
HIGV GP (KF848981)         53:TSWMRNNSAFTGKVGMYQGRDKVEYFPAEENFQWKWPGLLSPCDADWMGIIRVVPAPKGV 112
                               ** **   * *  *   **  * ***        * ******** * *   *  *

SFTS virus GP (AB817992)  835:RVDDAVCYSKITSVEAVANYSAIPTTIGGLRFERSHDSQGKISGSPLDITAIRGSFSVNY 894
GTV GP (NC_043609)        832:RVDDAVCFSKITSVEAVANYSAIPTIIGGLRFERSHDSQGKISGSPLDITAIRGEFSVSY 891
GTV GP (KT328592)         832:RVDDAVCFSKITSVEAVANYSAIPTIIGGLRFERSHDSQGKISGSPLDITAIRGEFSVSY 891
GTV GP (MH688509)         832:RVDDAVCFSKITSVEAVANYSAIPTIIGGLRFERSHDSQGKISGSPLDITAIRGEFSVSY 891
HRTV GP (MZ617375)        831:RVDDAVCFSKLTSVEAVANFSKIPATISGVRFDQGNHGESRIYGSPLDITRVSGEFSVSF 890
HRTV GP (MZ617372)        839:RVDDAVCFSKLTSVEAVANFSKIPATISGVRFDQGNHGESRIYGSPLDITRVSGEFSVSF 898
HRTV GP (MZ617369)        834:RVDDAVCFSKLTSVEAVANFSKIPATISGVRFDQGNHGESRIYGSPLDITRVSGEFSVSF 893
HRTV GP (KJ740147)        839:RVDDAVCFSKLTSVEAVANFSKIPATISGVRFDQGNHGESRIYGSPLDITRVSGEFSVSF 898
HRTV GP (NC_024494)       839:RVDDAVCFSKLTSVEAVANFSKIPATISGVRFDQGNHGESRIYGSPLDITRVSGEFSVSF 898
HRTV GP (JX005845)        839:RVDDAVCFSKLTSVEAVANFSKIPAIISGVRFDQGNHGESRIYGSPLDITKVSGEFSVSF 898
HRTV GP (JX005844)        839:RVDDAVCFSKLTSVEAVANFSKIPATISGVRFDQGNHGESRIYGSPLDITRVSGEFSVSF 898
HIGV GP (NC_027715)       828:RTDDITCYTKLTSVEAVANYSRVPCIMGGIRFERSPHERGRVIGSPTDITSVKGSFSVTF 887
HIGV GP (KM198926)        828:RTDDITCYTKLTSVEAVANYSRVPCIMGGIRFERSPHERGRVIGSPTDITSVKGSFSVTF 887
HIGV GP (KF848981)        828:RTDDITCYTKLTSVEAVANYSRVPCIMGGIRFERSPHERGRVIGSPTDITSVKGSFSVTF 887
                              * **  *  * ******** *  *    * **           *** ***   * ***

SFTS virus GP (AB817992)  895:RGLRLSLSEITATCTGEVTNVSGCYSCMTGAKVSIKLHSSKNSTAHVRCKGDETAFSVLE 954
GTV GP (NC_043609)        892:RGLRLSLSEITATCTGEVTNISGCYSCMMGAKVSIRLHSNKNSTAHLKCSSDETAFSVSE 951
GTV GP (KT328592)         892:RGLRLSLSEITATCTGEVTNISGCYSCMMGAKVSIRLHSNKNSTAHLKCSSDETAFSVSE 951
GTV GP (MH688509)         892:RGLRLSLSEITATCTGEVTNISGCYSCMMGAKVSIRLHSNKNSTAHLKCSSDETAFSVSE 951
HRTV GP (MZ617375)        891:RGMRLRLSEISASCTGEITNVSGCYSCMTGASVSIKLHSSKNTTGHLKCDSDETAFSVME 950
HRTV GP (MZ617372)        899:RGMRLRLSEISASCTGEITNVSGCYSCMTGASVSIKLHSSKNTTGHLKCDSDETAFSVME 958
HRTV GP (MZ617369)        894:RGMRLRLSEISASCTGEITNVSGCYSCMTGASVSIKLHSSKNTTGHLKCDSDETAFSVME 953
HRTV GP (KJ740147)        899:RGMRLKLSEISASCTGEITNVSGCYSCMTGASVSIKLHSSKNTTGHLKCDSDETAFSVME 958
HRTV GP (NC_024494)       899:RGMRLKLSEISASCTGEITNVSGCYSCMTGASVSIKLHSSKNTTGHLKCDSDETAFSVME 958
HRTV GP (JX005845)        899:RGMRLKLSEISASCTGEITNVSGCYSCMTGASVSIKLHSSKNTTGHLKCDSDETAFSVME 958
HRTV GP (JX005844)        899:RGMRLKLSEISASCTGEITNVSGCYSCMTGASVSIKLHSSKNTTGHLKCDSDETAFSVME 958
HIGV GP (NC_027715)       888:RGLRLKLSETMATCTGEFVNLTGCYSCMHGATAEFKISSNKNTTAHVVCEHDKTAFEVHE 947
HIGV GP (KM198926)        888:RGLRLKLSETMATCTGEFVNLTGCYSCMHGATAEFKISSNKNTTAHVVCEHDKTAFEVHE 947
HIGV GP (KF848981)        888:RGLRLKLSETMATCTGEFVNLTGCYSCMHGATAEFKISSNKNTTAHVVCEHDKTAFEVHE 947
                              ** ** ***  * ****  *  ****** **       * ** * *  *  * *** * *
```

S13 Fig: Alignment of amino acid sequences of the GP of SFTS virus and it’s related viruses

Amino acid sequences of GP of indicated viruses were aligned using GENETYX software. Accession numbers of sequences used are shown in parentheses. Regions including five N-linked glycosylation motifs of SFTS virus GP are shown. Red bolds are N-linked glycosylation motifs. GTV, Guertu virus; HRTV, Heartland virus; HIGV, Hunter Island group virus. Stars indicate conserved amino acids among the aligned sequences.
