# Supplementary material for: N-glycosylation of viral glycoprotein is a novel determinant for the tropism and virulence of highly pathogenic tick-borne bunyaviruses
Source: PLoS Pathog. 2024 Jul 15;20(7):e1012348. doi: 10.1371/journal.ppat.1012348 (PMC11271937; doi:10.1371/journal.ppat.1012348)
Supplement: S14 Fig — Mean body weight of inoculated AG129 mice (Fig 8) are shown. (PDF) [file ppat.1012348.s014.pdf]

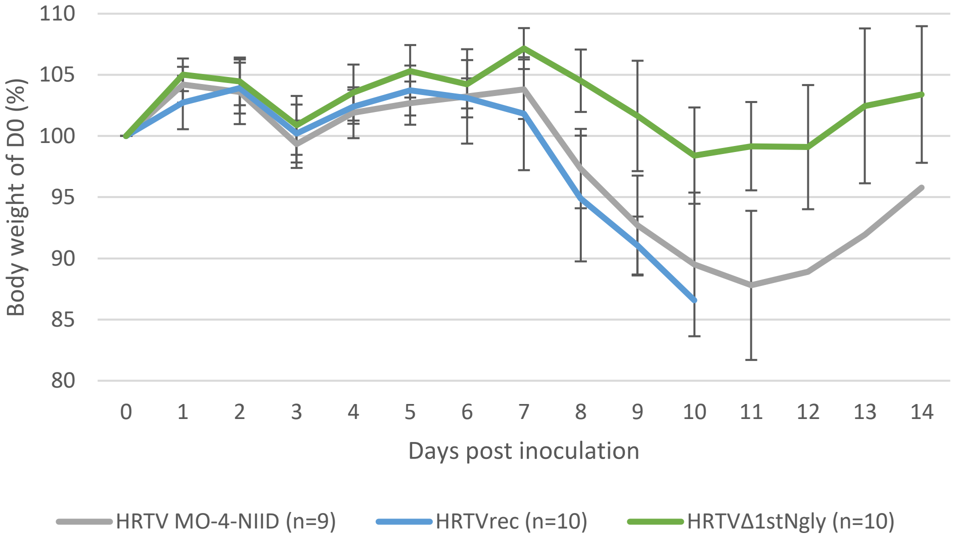

S14 Fig: Body weight of AG129 mice inoculated with the Heartland virus
Mean body weight of inoculated AG129 mice (Fig 8) are shown.
